# Multidimensional mechanisms of SFTSV-host interactions: viral protein functions, m^6^A modification, tick saliva factors, and platelet regulation

**DOI:** 10.3389/fcimb.2026.1827915

**Published:** 2026-09-14

**Authors:** Tao Wang, Meiyu Zheng, Wenhe Zhu, Xianmin Feng

**Affiliations:** 1 College of Medicine, Yanbian University, Yanji, Jilin, China; 2 School of Basic Medicine Sciences, Jilin Medical University, Jilin, China; 3 Jilin Provincial Key Laboratory of Changbai Mountain Authentic Medicial Materias’ Quality Assurance & Resources Development, Jilin, China

**Keywords:** autophagy, m^6^A modification, platelet, severe fever with thrombocytopenia syndrome virus, tick saliva peptide HIDfsin2

## Abstract

Severe fever with thrombocytopenia syndrome virus (SFTSV), officially designated as Dabie bandavirus by the International Committee on Taxonomy of Viruses (ICTV), is an emerging zoonotic pathogen belonging to the order Bunyavirales and the family Phenuiviridae. First isolated and identified in 2010 from the serum of patients with severe fever with thrombocytopenia syndrome (SFTS) in China, SFTSV has since been reported in multiple provinces across China, as well as in South Korea, Japan, Vietnam, the United Arab Emirates (including Dubai), and Pakistan. With a case fatality rate of up to 30%, SFTSV poses a serious threat to public health and carries the potential for broader global spread. In nature, SFTSV is primarily transmitted through tick bites, although direct human-to-human or animal-to-human transmission can also occur through exposure to the blood, respiratory secretions, or other bodily fluids of infected individuals or animals. The pathogenic mechanisms of SFTSV are complex and multifaceted, involving the coordinated actions of multiple viral proteins, host epigenetic regulation, and virus–vector interactions. In this review, we take tick bites as the starting point to describe how SFTSV achieves cross-species transmission through ticks, summarize the viral life cycle, including internalization, replication, assembly, and egress, and discuss the antagonistic interplay between the viral life cycle and host defense responses, with the aim of providing a solid theoretical foundation for understanding the infection and pathogenesis of SFTSV.

## 1 Introduction

SFTSV is a segmented negative-sense RNA virus with spherical or pleomorphic virions approximately 80–100 nm in diameter, characterized by a 5–7 nm-thick lipid bilayer envelope bearing 5–10 nm-long surface spikes composed of the glycoproteins Gn and Gc (Jia et al., 2024), and an internal ribonucleoprotein (RNP) complex formed by genomic RNA tightly encapsidated by the nucleoprotein (Np) (Li, 2024). During the infection process, the virus releases the RNP into the host cell cytoplasm via membrane fusion, and its key life-cycle events, including genome transcription, translation, and virion assembly, are all concentrated and completed within the cytoplasm. The SFTSV genome consists of three RNA segments, designated large (L), medium (M), and small (S), which encode the RNA-dependent RNA polymerase (RdRp), the envelope glycoprotein precursor (Gn/Gc), and both the nucleoprotein (Np) and nonstructural protein (NSs), respectively (Liu et al., 2014; Lee et al., 2023). Collectively, these virus-encoded proteins construct a regulatory network for SFTSV infection across three major stages: host cell entry, genome amplification, and immune evasion, thereby creating an immune-sheltered microenvironment for viral replication (**Figure 1**).

**Figure 1:**
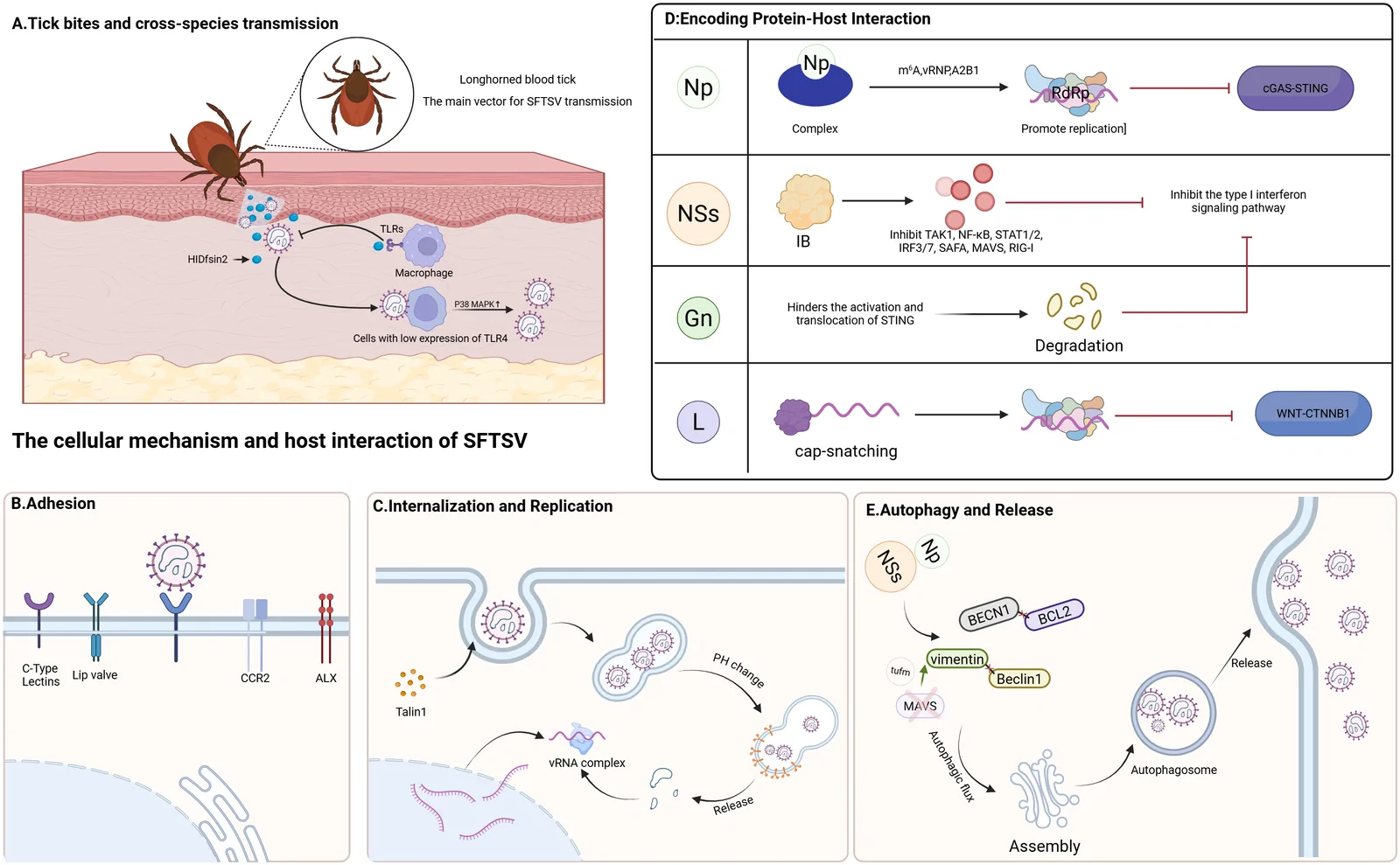
Overview of SFTSV-host interactions and the viral life cycle. **(A)** Tick bite and cross-species transmission. Haemaphysalis longicornis serves as the primary vector for SFTSV transmission. Upon tick attachment to the host's skin, the virus and tick salivary factors are co-released into the localized microenvironment. Host macrophages participate in viral recognition via pattern recognition receptors such as TLRs; however, in cells with low TLR4 expression, the virus more readily establishes infection and activates relevant signaling pathways (e.g., p38 MAPK) to promote viral dissemination. **(B)** Viral attachment. SFTSV initially binds to the host cell via surface receptors or attachment factors, including C-type lectins, lipid rafts, CCR2, and AXL, which collectively mediate viral attachment and initiate the entry process. **(C)** Endocytosis and replication. The virus undergoes receptor-mediated endocytosis to enter the host cell. Driven by endosomal acidification (pH change), the virus completes membrane fusion and uncoating, releasing the viral ribonucleoprotein (vRNP) complex into the cytoplasm for subsequent genome replication and transcription. **(D)** Interactions between virus-encoded proteins and the host. Distinct SFTSV proteins modulate host antiviral responses through multiple mechanisms: Np interacts with host factors to promote viral replication and inhibits the cGAS-STING signaling pathway. NSs induces the formation of inclusion bodies (IBs) to sequester or suppress key molecules, including TBK1, NF-κB, STAT1/2, IRF3/7, SAFA, MAVS, and RIG-I, thereby effectively abrogating the type I interferon signaling pathway. Gn impedes the activation and translocation of STING and facilitates the degradation of related components. The L protein executes viral RNA transcription via a "cap-snatching" mechanism and extensively associates with host pathways, such as the WNT-CTNNB1 pathway. **(E)** Autophagy and viral release. SFTSV intricately regulates the host autophagic flux. By exploiting molecules such as NSs, NP, BECN1, BCL2, vimentin, Beclin1, and MAVS, the virus promotes autophagosome formation, viral assembly, and maturation, ultimately orchestrating the egress of progeny virions via autophagy-dependent pathways.

## 2 Role of tick salivary factors in SFTSV

Arboviruses are a group of viruses transmitted by blood-sucking arthropods, with mosquitoes and ticks serving as the primary vectors. Representative pathogens include dengue virus, Chikungunya virus, Japanese encephalitis virus, West Nile virus, and severe fever with thrombocytopenia syndrome virus (SFTSV) (Harsh and Eleftherianos, 2020; Laureti et al., 2020). In recent years, climate change and ecological alterations have continuously expanded the geographical distribution of these vectors, increasing the risk of cross-regional pathogen transmission and thereby making arboviral diseases one of the major public health threats worldwide (Cholvi et al., 2024).

During the establishment of early infection, a complex and dynamic interplay exists between ticks, tick-borne pathogens, and the host immune system. The host skin serves as both the primary barrier against viral invasion and the principal portal of entry for these pathogens (Hermance and Thangamani, 2020). Mechanical injury caused by tick attachment rapidly triggers the host’s wound-healing programs, predominantly characterized by hemostasis and inflammation, which can impede sustained tick feeding (Esteves et al., 2025). Consequently, ticks have evolved sophisticated regulatory strategies mediated by salivary components to counteract these repair responses and remodel the local microenvironment at the bite site. This modulation creates favorable conditions for both blood-feeding and pathogen dissemination, thereby facilitating successful viral colonization within the host.

Current studies indicate that active components in tick saliva facilitate pathogen transmission primarily through two distinct pathways: anti-hemostasis and immunomodulation (Tirloni et al., 2017). For instance, regarding anti-hemostatic functions, apyrase hydrolyzes ATP and ADP to inhibit platelet aggregation (Perumalsamy et al., 2024); tick salivary gland protein 3 (TSGP3) suppresses collagen-platelet binding and its interaction with thromboxane A2, thereby preventing both platelet aggregation and vasoconstriction; meanwhile (Beaufays et al., 2008), TIX-5 targets coagulation-related factors to hinder the coagulation cascade (Šimo et al., 2017). In terms of immunomodulation, Salp15, lipocalin proteins, and other salivary gland-derived proteins can dampen dendritic cell signaling, thereby attenuating the host immune response (Carvalho-Costa et al., 2015; Pham et al., 2021). Concurrently, an IL-2-binding protein suppresses T cell proliferation, while B-cell inhibitory factor (BIF) halts B cell activation and expansion (Pham et al., 2021). Furthermore, during tick feeding and pathogen transmission, salivary gland extract (SGE) can antagonize local angiogenesis and immune responses by inhibiting TGF-β and ERK signaling pathway-mediated fibroblast migration (Bartíková et al., 2020). SGE also directly impairs the motility of macrophages and monocytes and alters cytokine production, further facilitating tick feeding and pathogen dissemination (Schneider et al., 2021). Similarly, in the context of mosquito-borne viruses, SGE has been shown to downregulate inflammasome activation induced by Dengue and Zika virus infections, effectively suppressing innate immunity and promoting viral replication (Shrivastava et al., 2024). Collectively, these effects contribute to the establishment of a localized immune-privileged microenvironment highly conducive to pathogen colonization. It is worth noting, however, that while the aforementioned factors represent the general paradigm of how tick saliva modulates host hemostasis and immunity, the precise mechanisms linking specific salivary components directly to the transmission of select pathogens remain incompletely resolved. Importantly, a recent study has revealed that HIDfsin2, a salivary factor derived from SFTSV-infected ticks, plays a pivotal role in mediating SFTSV transmission.

Specifically, The tick salivary factor HIDfsin2 exerts a bidirectional regulatory effect during SFTSV infection. Upon recognition of HIDfsin2, Toll-like receptor 4 (TLR4) on the surface of macrophages triggers a robust innate immune response, effectively suppressing SFTSV replication. Mechanistically, this process hinges on the interaction between HIDfsin2 and lipopolysaccharide (LPS), where HIDfsin2 disrupts the micellar structure of LPS, thereby upregulating TLR4 expression and activating downstream signaling cascades. Specifically, it induces the activation of the NF-κB signaling pathway and the nuclear translocation of the p38 protein. This intricate regulation culminates in the explosive release of downstream inflammatory cytokines and type I interferons (IFNs), collectively forging a robust barrier against viral infection (Wang L. et al., 2024). However, in stark contrast to this TLR4-mediated antiviral mechanism, in cells with low TLR4 expression (such as A549 and Huh7), HIDfsin2 aberrantly amplifies p38 MAPK activation via the upstream MKK3/6 kinase pathway (Wang et al., 2023). This creates a highly permissive environment for SFTSV replication, elegantly illustrating how the virus exploits the complexity of host signaling pathways to facilitate its own propagation. This phenomenon indicates that tick salivary factors do not exert a strictly unilateral promotive or suppressive effect on SFTSV infection; rather, their ultimate impact is highly contingent upon the host cell type and the specific expression profile of pattern recognition receptors (PRRs). Taken together, tick saliva functions not merely as an accessory tool for vector blood-feeding, but acts as a crucial regulatory node bridging the tick, the virus, and the host immune response.

## 3 Interactions between SFTSV-encoded proteins and host cells

### 3.1 Mechanisms of receptor recognition and membrane fusion mediated by Gn/Gc glycoproteins

SFTSV cellular entry is initiated by the binding of Gn/Gc to the cell surface receptor (DC-SIGN) and attachment factors (HS, NMMHCIIA) (Hicks et al., 2024). Both glycoproteins are derived from a glycoprotein precursor (GP) encoded by the viral M segment. Within the endoplasmic reticulum (ER) lumen, GP is cleaved and processed by host proteases to generate two functionally complementary mature subunits: the N-terminal glycoprotein (Gn) and the C-terminal glycoprotein (Gc). On the viral envelope surface, they exist as heterodimers (Moming et al., 2021), and further assemble into pentamers and hexamers (Sun Z. et al., 2023).

The type I transmembrane protein Gn serves as the primary spike structure on the virion surface, mediating viral attachment and endocytosis by specifically binding to host receptors via its highly glycosylated N-terminal domain (Ren et al., 2024). Beyond the previously reported receptor DC-SIGN, subsequent investigations into alternative entry pathways have identified C-C motif chemokine receptor 2 (CCR2) and pro-low-density lipoprotein receptor-related protein 1 (LRP1) as additional critical factors for viral entry. Regarding the former, CCR2 exists as two splice isoforms, CCR2A and CCR2B, which differ in their C-terminal lengths and localize to the plasma membrane. Both isoforms interact directly with Gn through tyrosine sulfation at the Y26 residue of the N-terminal domain (Zhang L. et al., 2023). This interaction is particularly significant because CCR2 is highly expressed in monocytes and macrophages, identifying these as primary target cells for SFTSV infection (Ahmad et al., 2022); furthermore, as a chemokine receptor, CCR2 facilitates the recruitment of inflammatory monocytes, where its binding with Gn triggers pro-inflammatory effects that may correlate with clinical severity (Park and Kim, 2026). In parallel, LRP1 has also been demonstrated as a crucial host factor during the viral entry stage, binding directly to Gn via its CLI and CLII motifs (Xing et al., 2025). Together, these receptor-mediated mechanisms provide clarity to the long-standing debate regarding the target organs and cells of SFTSV. While traditional views attributed multi-organ damage to systemic inflammation induced by B-lineage lymphocyte infection, recent evidence reveals that SFTSV can directly infect pancreatic tissue via CCR2 and LRP1. This direct infection triggers localized immune responses and tissue damage (Liu et al., 2026), offering a new perspective on SFTSV-induced multi-organ dysfunction and suggesting that similar direct infection mechanisms may occur in other organs.

In addition to the aforementioned traditional receptors, recent studies have identified AXL as another critical entry receptor for SFTSV. Identified through genome-wide CRISPRa screening, AXL significantly enhances SFTSV infection and can synergistically facilitate viral entry into host cells when co-expressed with DC-SIGN. Unlike CCR2 and LRP1, which primarily rely on the direct recognition of Gn, AXL-mediated viral entry depends more on an “apoptotic mimicry” mechanism. Specifically, phosphatidylserine (PS) on the viral envelope binds to AXL via the bridging ligand GAS6, forming a “PS-GAS6-AXL” complex. This complex subsequently induces the autophosphorylation of intracellular tyrosine residues on AXL, recruiting PI3K and PLC to activate the PI3K/PLC-dependent macropinocytosis pathway, thereby accelerating viral internalization. Notably, this mechanism remains effective in DC-SIGN-negative cells, such as HUVECs, suggesting that AXL might serve as a crucial entry factor for the early dissemination of SFTSV in tissues like skin and endothelium. Furthermore, these findings indicate that SFTSV is capable of adopting distinct receptor recognition and endocytic strategies depending on diverse cellular contexts (Jin et al., 2025).

Following the initial binding of Gn to cell surface receptors, SFTSV can be internalized into host cells via multiple endocytic mechanisms, including clathrin-mediated endocytosis, lipid raft/caveolin-dependent pathways, and macropinocytosis (Yu Y. et al., 2025). Among these mechanisms, lipid rafts play a well-documented role in facilitating the cellular entry of numerous enveloped viruses, such as influenza virus (IV), herpes simplex virus type 1 (HSV-1), human immunodeficiency virus (HIV), Ebola virus (EBOV), Marburg virus (MARV), and Epstein-Barr virus (EBV) (Takahashi and Suzuki, 2011; Amini-Bavil-Olyaee et al., 2013). During SFTSV infection, upon binding to lipid rafts, the viral glycoprotein Gn recruits the TALIN1 protein into these lipid rafts. This leads to a specific interaction between TALIN1 and Gn, thereby mediating the viral internalization process (Cheng et al., 2024).

Once internalized via endocytosis, SFTSV is encapsulated within vesicular structures and sequentially transported to Rab5+ early endosomes and Rab7+ late endosomes. As the late endosomes gradually acidify (pH ≈ 5.6) (Shen et al., 2022), the class II fusion protein Gc undergoes a pH-dependent conformational rearrangement, thereby driving the fusion of the viral envelope with the host endosomal membrane (Du et al., 2023). During the membrane fusion stage, the fusion peptide of the Gc protein is inserted into the target host membrane; under acidic conditions, the protein shifts from a dimer to a trimer (Jeon et al., 2024). This conformational change shortens the distance between the viral envelope and the endosomal membrane, forcing the two membranes to fuse and thereby releasing the viral genome into the cytoplasm (Jeon et al., 2024). Furthermore, an increasing number of studies indicate that Gn/Gc are not solely involved in viral entry processes—such as attachment, endocytosis, and membrane fusion—but also participate in immune evasion by interfering with host innate immune signaling pathways.

### 3.2 Mechanisms of immune evasion mediated by Gn/Gc

SFTSV infection of host cells leads to the release of mitochondrial DNA (mtDNA) into the cytoplasm, which is subsequently captured by the DNA sensor cyclic GMP-AMP synthase (cGAS), thereby activating the innate immune response mediated by the cGAS-STING signaling pathway (Sun et al., 2025a). The molecular mechanism of this pathway primarily involves the following key steps: upon binding to mtDNA via its N-terminal domain, cGAS undergoes a conformational change to form a 2:2 dimeric configuration and exposes its enzymatic active site. It subsequently catalyzes the synthesis of the second messenger cGAMP from ATP and GTP; cGAMP then specifically binds to the ligand-binding domain of the endoplasmic reticulum (ER) membrane protein STING (Jiang et al., 2024). In the resting state, STING is anchored to the ER membrane as a dimer via its C-terminal transmembrane domain, with its carboxy-terminal dimerization domain (DD) forming the ligand-binding pocket (Guimarães et al., 2021; Zheng and Gao, 2023). The binding of cGAMP induces the oligomerization of STING and triggers its translocation across the ER-Golgi-perinuclear membrane system. During this translocation process, STING recruits TANK-binding kinase 1 (TBK1) via its C-terminal tail (CTT) domain, forming the STING-TBK1 signaling complex (Sun C. et al., 2023). The activation of TBK1 triggers its kinase activity, which activates the IRF3 transcription factor through a cascade of phosphorylation and ubiquitination modifications. Following nuclear translocation, phosphorylated IRF3 synergizes with nuclear factor-kappa B (NF-κB) to act on the promoter region of type I interferon genes, ultimately mediating the secretion of antiviral cytokines (Wang B. et al., 2022). The SFTSV glycoproteins Gn/Gc can antagonize this pathway through multiple mechanisms: after completing dimerization in the ER, Gn translocates to the Golgi apparatus, where its C-terminal domain (amino acids 1-200) interacts with the CTT domain of STING (amino acids 196-340) (Jiang et al., 2024). This interaction directly interferes with the formation of STING dimers, thereby blocking its oligomerization and subsequent translocation process. Additionally, Gn specifically inhibits the K27 ubiquitination of STING, significantly reducing its ability to recruit TBK1. Furthermore, during the late stages of infection, Gn further attenuates the host antiviral response by promoting the autophagic degradation of STING (Jiang et al., 2024) (**Figure 2**).

**Figure 2:**
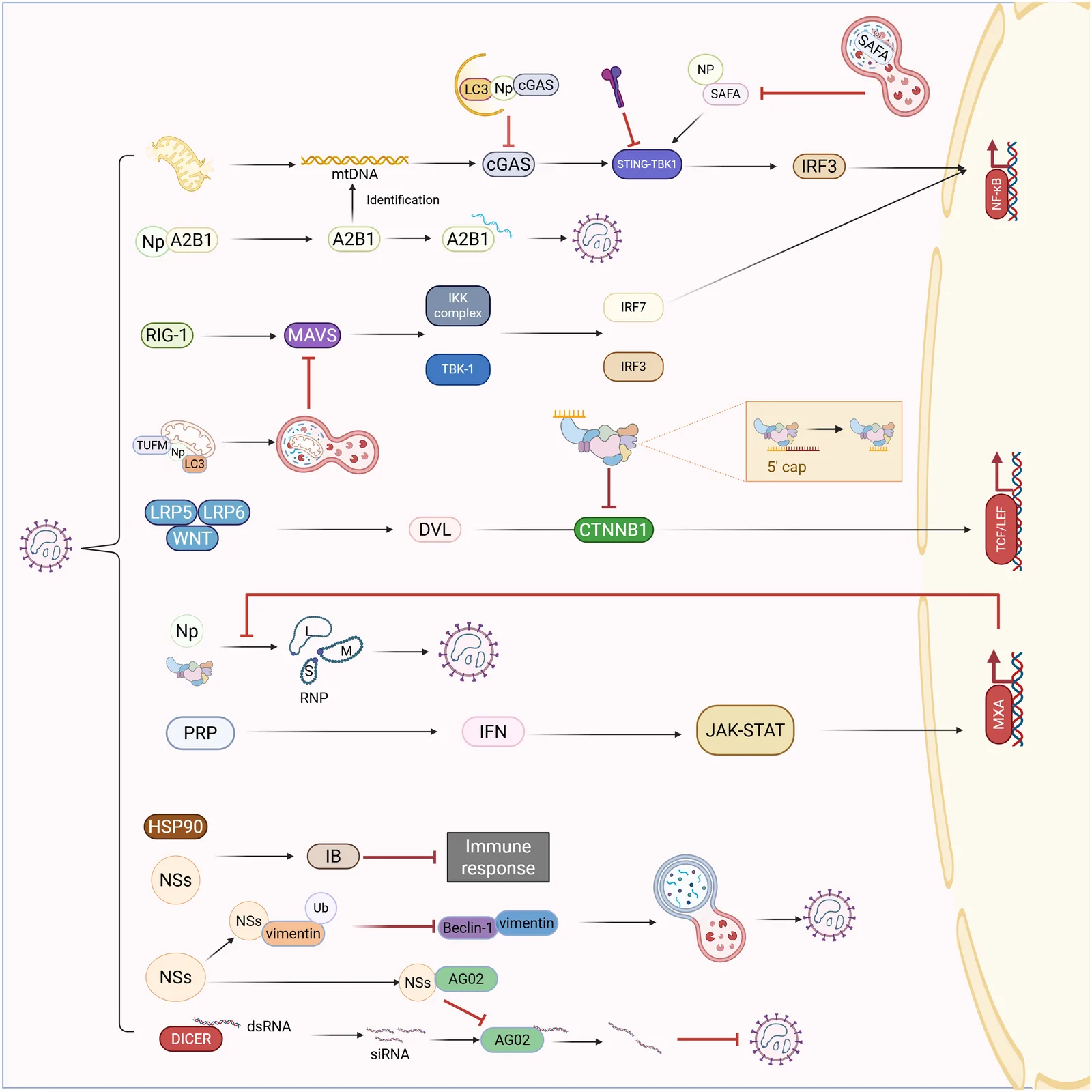
Mechanisms of SFTSV immune evasion. Regulatory mechanisms of Severe Fever with Thrombocytopenia Syndrome Virus (SFTSV) on innate immune recognition, interferon production, and downstream antiviral effector pathways following host cell infection. Under conditions of mitochondrial damage or infection-induced stress, mitochondrial DNA (mtDNA) is released into the cytoplasm and recognized by cGAS, activating the STING-TBK1-IRF3 signaling axis to induce the expression of type I interferons (IFNs) and related antiviral genes. Concurrently, the RIG-I-MAVSTBK1/ IKK-IRF7 pathway is involved in viral RNA recognition and the activation of consecutive immune responses. SFTSV proteins intervene in host antiviral responses via multiple cellular targets. NP interacts with host factors such as A2B1 and SAFA to suppress cGAS-STING signaling and promote viral ribonucleoprotein (RNP) replication. Additionally, the virus modulates the WNTDVL- CTNNB1 pathway to influence host transcriptional responses. On the other hand, NSs induces the formation of inclusion bodies (IBs) to suppress host immune responses, and impacts autophagy as well as RNA interference (RNAi) processes by regulating molecules such as vimentin, Beclin1, and AGO2. Meanwhile, the virus interferes with PRR-IFN-JAK-STAT signal transduction, suppressing the expression of antiviral effector genes such as MxA, IFITM3, and NF-κB. Overall, SFTSV achieves robust immune evasion and facilitates viral replication and release by dampening pathogen recognition, intercepting interferon signaling, and remodeling intracellular homeostasis.

### 3.3 The role of L protein in viral replication

After entering host cells via endocytosis, the virus completes membrane fusion under the influence of late endosomal acidification and releases its genome into the cytoplasm. Subsequently, SFTSV rapidly initiates the transcription and replication processes. This process is primarily mediated by the RNA-dependent RNA polymerase (RdRp) encoded by the L segment, namely the L protein, whose function relies on the synergistic coordination of multiple domains, including the core catalytic domain, the cap-binding (CapB) domain responsible for recognizing the 5’ cap structure of host mRNAs, and the Endo domain with endonuclease activity (Kim and Park, 2023; Kim D. et al., 2024). During genome replication, the virus first uses the negative-sense genomic RNA (vRNA) as a template to synthesize positive-sense complementary RNA (cRNA), and subsequently uses the cRNA as a template to generate new progeny vRNAs (Williams et al., 2023).

Viral genome replication primarily proceeds via two pathways, namely replicating from both termini or initiating from the internal generation of short primers (Williams et al., 2024). Current structural data on how the SFTSV L protein facilitates viral genome replication indicate that SFTSV genome replication tends towards the realignment of short primers, initiating replication via a mechanism known as”cap-snatching” (Vogel et al., 2020). The CapB domain of the L protein binds and cleaves host capped RNAs to serve as viral replication primers; related studies show that RNAs associated with the WNT-CTNNB1 signaling pathway in host cells may become an important “reservoir” for viral transcription initiation primers, thereby triggering the downregulation of this signaling pathway (Jiang et al., 2023). This phenomenon is not limited to SFTSV; a similar downregulation trend in the WNT-CTNNB1 signaling pathway has also been observed during infections with other segmented negative-sense RNA viruses, such as Lassa virus (LASV) and influenza A virus (IAV) (Jiang et al., 2023). In the context of viral infection, the L protein interacts with WNT pathway-associated RNAs, utilizing CapB to capture host mRNAs, while the Endo domain cleaves short RNA fragments at the 5’ ends of host cell mRNAs, snatching the host mRNA caps to serve as primers for transcription initiation. Subsequently, the cap-snatched WNT pathway-associated RNAs undergo degradation, ultimately causing a decline in overall CTNNB1 levels and thereby affecting the activation of downstream transcription factors (Jiang et al., 2023), a cascade of events that ultimately leads to disease exacerbation or even death. Studies indicate that the WNT-CTNNB1 pathway exerts a certain regulatory role in platelet function, including aspects such as platelet adhesion, aggregation, and activation (Woldeamanuel et al., 2025), whereas alterations in platelet function and reductions in platelet count are typical hallmarks of human SFTSV infection, and this pathway along with its downstream transcription factors may contribute to impaired T cell responses (Dai et al., 2016). The expression level of this pathway holds promise as a biomarker for evaluating disease prognosis, possessing value for further investigation.

After completing cap-snatching, the viral genome then begins to initiate viral replication dependent on the conformational changes of the L protein. Cryo-electron microscopy (cryo-EM) further resolved five conformational states of the L protein during the replication process from pre-initiation to the late-elongation stage (Williams et al., 2023). In the initiation stage, the L protein binds with the 5’cRNA and undergoes conformational change; in the dynamic conformations of the viral polymerase, the vRNA-binding lobe (vRBL) coordinately rearranges with the adjacent fingers domain and the PA-C-like core lobe, forming a binding interface rich in positive charges to specifically capture the 5’ end RNA, namely the 5’ hook structure, and subsequently the L protein enters the elongation stage. The elongation stage can be divided into three subtypes based on differences in endonuclease conformations: “Early-elongation”, “Early-elongation-endo”, and “Late-elongation”. Therein, the Endo domain completes the terminal stage of elongation through a “protruding” conformational transition, at which point the distal double strand forms a stable 3’RNA and guides it into the RdRp active center of the L protein, accommodating a 10bp product-template duplex structure within the RdRp core, thereby starting the replication process. Finally, the 3’ and 5’ ends of the cRNA bind with the hook binding site and the secondary binding site, respectively; this stage does not form a distal duplex structure, and the L protein enters a resting conformation (**Figure 2**).

### 3.4 Viral RNA protection and immune evasion functions of the Np

Upon the release of the viral genome into the cytoplasm, the nucleocapsid protein Np wraps the viral RNA in an exquisite hexameric/pentameric ring structure and forms a functional conjugate with the L protein, jointly constituting the viral ribonucleoprotein complex (vRNP) (Wang et al., 2025). This unique morphology serves not only as a protective shell for the viral genetic material, but also as a core architecture actively participating in the viral replication and assembly processes. This encapsulation strategy of the Np protein can effectively resist degradation by host cellular nucleases and attacks from the host immune system, thereby ensuring the integrity and stability of the viral genome (Park et al., 2025). This encapsulation strategy possesses evolutionary conservation among viruses of the Phenuiviridae family; further analysis of this phenomenon helps to deepen the understanding of viral immune evasion mechanisms and propel the development of new antiviral drugs.

Np can hijack host factors to promote replication. Heterogeneous ribonucleoprotein A2B1 (hnRNP A2B1), as a core member of the eukaryotic hnRNP complex, participates in the replication process of various RNA viruses through a nucleocytoplasmic transport reprogramming mechanism, and its function possesses significant evolutionary conservation (Zhang et al., 2024). For example, during infections with Sendai virus (SeV), vesicular stomatitis virus (VSV-GFP), enterovirus 71 (EV71), and Zika virus (ZIKV), it exhibits translocation from the nucleus to the cytoplasm (Zhang et al., 2024). In SFTSV infection, Np directly interacts with the RRMs domain of A2B1, competitively blocking its nuclear localization signal (NLS) function and mediating the retention of hnRNP A2B1 in the cytoplasm; the latter interacts with the 5’ UTR of SFTSV RNA segments to enhance SFTSV replication (Zhang et al., 2024).

Np Participates in Virus-Host Interactions Through the Type I Interferon Response. Studies have shown that the host heterogeneous ribonucleoprotein A2B1 (hnRNP A2B1) is involved in sensing mitochondrial DNA (mtDNA) leakage during SFTSV infection and triggers the STING-TBK1 axis to enhance the type I interferon response (Huang et al., 2025). By capturing newly synthesized hnRNP A2B1 in the cytoplasm, SFTSV NP recognizes mitochondrial mtDNA leakage, and subsequently mediates the activation of the STING-TBK1 signaling pathway to promote IFN-β production. These findings reveal the role of hnRNP A2B1 in sensing SFTSV infection in the cytoplasm and activating innate immunity. This suggests that by targeting hnRNP A2B1, novel antiviral therapies against SFTSV and other RNA viral infections can potentially be developed.

Recent studies have also revealed that nuclear scaffold attachment factor A (SAFA) is involved in host innate immune recognition during SFTSV infection. In the resting state, SAFA is primarily localized in the nucleus; however, following SFTSV infection, its expression level is upregulated, and it is retained in the cytoplasm mediated by the viral nucleocapsid protein Np (Amurri et al., 2023). This process relies on the binding of the viral nucleocapsid protein Np to the nuclear localization signal (NLS) domain of SAFA. The cytoplasm-retained SAFA recognizes exogenous SFTSV RNA, binding specifically to the viral S- and M-segment RNAs; on this basis, SAFA further recruits and promotes the activation of the STING-TBK1 signaling axis, enhancing the phosphorylation levels of TBK1, IRF3, and NF-κB related pathways, thereby inducing the expression of type I interferons and various pro-inflammatory cytokines, and ultimately inhibiting SFTSV replication (Amurri et al., 2023). Furthermore, host cell Myxovirus Resistance Protein A (MxA) specifically targets the N-terminal domain of Np in an RNA-independent manner, blocking the Np-RdRp interaction and interfering with RNP activity, which in turn inhibits viral replication (Chang et al., 2024; Li, 2024). However, Np is not merely a passive target of host antiviral recognition; it also possesses significant immune evasion functions. Studies have demonstrated that Np specifically binds to the 161–382 domain of cyclic GMP-AMP synthase (cGAS) to form an Np-cGAS-LC3 ternary complex. This complex is directionally transported to the lysosomal system for degradation via the autophagy pathway, successfully inhibiting the activation of the cGAS-STING signaling pathway and significantly weakening the host type I interferon response, thereby facilitating the establishment of an intracellular microenvironment conducive to the virus (Jiang et al., 2024).

Np as a Crucial Diagnostic Target and Protective Vaccine Immunogen. Owing to its high conservation across diverse RNA viruses, the nucleocapsid protein Np serves as the premier diagnostic target for SFTSV, underpinning the successful development of various Np-specific monoclonal antibodies, ELISA assays, and S-segment-targeted PCR tools (Kim S. et al., 2024; Stamper et al., 2025). Beyond its diagnostic utility, Np exhibits robust immunogenicity, eliciting potent Np-specific humoral and cellular immune responses following viral infection. Notably, recent subunit vaccine evaluations in IFNAR-KO mouse models demonstrated that a single-dose Np vaccination yielded a remarkable 66.7% survival rate against lethal SFTSV challenge, vastly outperforming the Gn subunit vaccine (which offered only a 16.7% survival rate), while concurrently and significantly boosting antigen-specific T-cell responses. Although Np-specific antibodies lack direct *in vitro* neutralizing activity, they play an indispensable collective role in *in vivo* antiviral immunity, likely acting in synergy with Np-specific T-cell responses to robustly enhance the overall protective efficacy (Kim S. et al., 2024). This paradigm of non-neutralizing yet protective Np-mediated immunity represents a conserved feature shared with other bunyaviruses; previous investigations have established that active or passive immunization targeting Np confers at least partial protection in animal models against lethal challenges of Rift Valley fever virus (RVFV) and Crimean-Congo Hemorrhagic Fever Virus(CCHFV) (Jansen van Vuren et al., 2011; Tipih and Burt, 2020). Consequently, although the detailed humoral mechanisms of these non-neutralizing antibodies remain to be fully elucidated, Np has emerged as an indispensable protective immunogen for the design of future SFTSV vaccines (**Figure 2**).

### 3.5 Immune evasion and viral RNA protection by the non-structural protein NSs

The Non-structural Protein NSs Inhibits Host Antiviral Immunity. As a crucial SFTSV virulence factor, its strategic mechanism lies in inducing the formation of a specialized lipid-body structure—the inclusion body (IB)—to hijack a series of key immune signaling molecules. These hijacked molecules include TANK-binding kinase 1 (TBK1), inhibitor of nuclear factor-κB (IκB) kinase subunit epsilon (IKKϵ), retinoic acid-inducible gene I (RIG-I), transcription factors STAT1 and STAT2, and interferon regulatory factors IRF3 and IRF7, thereby effectively suppressing the host immune response (Ning et al., 2019; Lee and Shin, 2021; Li et al., 2022). Studies have demonstrated that heat shock protein 90 beta (HSP90β) is involved in the formation and stabilization of NSs-mediated inclusion bodies (IBs) (Wang B. et al., 2024). Given the critical roles of the HSP90 family in protein folding, complex assembly, and functional maintenance across various viral life cycles (e.g., during dengue virus entry and herpes simplex virus transcription) (Kamal et al., 2004; Kubota and Hashiguchi, 2021; Ben Abdallah et al., 2024), focusing on the connection between HSP90 and SFTSV infection is of great significance. Particularly, interfering with the NSs-HSP90β interaction holds promise as a potential antiviral strategy to inhibit IB formation and restore the host immune response. Furthermore, NSs interacts with SAFA to mediate its nucleocytoplasmic translocation. By enhancing the interactions among SAFA, LC3, and SQSTM1, NSs subsequently drives the autophagic degradation of SAFA, downregulates the phosphorylation of TBK1 and p65, and thereby suppresses the host innate immune response (Yu TM. et al., 2025). Concurrently, the formation of IBs allows the Np-SAFA complex to be sequestered within these structures, effectively blocking the STING signal transduction pathway through spatial isolation to facilitate immune evasion (Huang et al., 2026). Nevertheless, whether the signaling mechanisms underlying the retention of SAFA by Np and NSs predominantly represent a viral immune evasion strategy or a host antiviral defense mechanism requires further investigation.

In addition to suppressing innate immune responses by sequestering pattern recognition receptor (PRR)-associated molecules within IBs and blocking subsequent signal transduction, NSs also protects viral RNA and promotes replication by antagonizing the RNA interference (RNAi) mechanism. As a core eukaryotic antiviral defense mechanism, RNAi utilizes small RNAs to mediate complementary sequence recognition, leading to target mRNA degradation or translational inhibition (Ying et al., 2007; Chable-Bessia et al., 2009). This pathway is initiated by the recognition of viral double-stranded RNA (dsRNA) by DICER, which processes it into multiple small interfering RNA (siRNA) duplexes (Bocos-Asenjo et al., 2022). NSs can interact with several components of the RNAi pathway, including AGO2, DICER, TNRC6A, and PRKRA, with a particularly noteworthy interaction with AGO2. NSs competitively binds to AGO2, primarily in an RNA-independent manner, thereby inhibiting the degradation of viral RNA by the RNAi machinery and facilitating viral replication (Javaid et al., 2025) (**Figure 2**).

## 4 Dual regulatory roles of m^6^A modification in SFTSV infection

Beyond direct viral-host protein interactions, epitranscriptomic regulation mediated by RNA chemical modifications represents another vital mechanism by which viruses intricately fine-tune gene expression and modulate host antiviral responses. With the continuous advancement of high-throughput sequencing technologies, over 170 distinct types of RNA chemical modifications have been identified in eukaryotic cells (Zhang et al., 2020). Collectively, these modifications construct a highly intricate epitranscriptomic regulatory network; by dynamically governing fundamental processes such as RNA splicing, nuclear export, transcript stability, translational efficiency, and immune evasion/recognition, this network plays an essential role in driving development, immune responses, and disease progression (Shi et al., 2020; Yang et al., 2024). Multiple studies have shown that many chemical modifications, originally found in eukaryotic cell RNAs, also exist in viral genomic RNAs and viral transcripts (Lichinchi et al., 2016; Courtney et al., 2017; Shen and Zhang, 2023). After the virus enters host cells, viral RNA can not only utilize the host’s chemical modifications to regulate its life cycle, but can also regulate the expression levels of host chemical modifications, greatly affecting viral replication and the host innate immune response (Gokhale et al., 2016; Lichinchi et al., 2016; Iwanami et al., 2020). In the exploration of the interactions between viruses and chemical modifications to date, researchers have found that only a very few chemical modifications play a role in virus-host interactions, such as N^7^-methylguanosine (m^7^G), 2′-O-methylated ribonucleosides (Nm), m^6^A, and 5-methylcytidine (m^5^C) (Williams et al., 2019; Furuse, 2021; Ma et al., 2021; Zhang et al., 2021).

N^6^-methyladenosine (m^6^A), as the most abundant dynamic and reversible post-transcriptional modification in mRNA, is coordinately regulated by methyltransferases (methylases), demethylases (demethylases), and reader proteins, forming a “Writer-Eraser-Reader” tripartite molecular regulatory axis (Yang et al., 2018; Ma et al., 2021). It plays an important role in the specific gene expression and replication of various pathogenic viruses, as well as the innate immune responses generated after viral infection of the host.

Multiple studies have shown that m^6^A modification is a dynamic and variable process, and there exist multiple modification patterns for m^6^A (Zhang et al., 2022). While m^6^A modification exerts a promotive effect on the replication of viruses such as SARS-CoV-2, IAV, HIV-1, EBV, and EV71, it exhibits inhibitory effects on the replication of HSV, KSHV, and various viruses within the Flaviviridae family (Zhang et al., 2021; Yanagi et al., 2022; Cao et al., 2024). Taking HBV as an example: on one hand, YTHDF2/YTHDF3 proteins reduce the stability of HBV RNA by binding to m^6^A modification sites at the 3′ stem-loop end, thereby affecting its protein expression; on the other hand, m^6^A modification at the 5′ end of HBV pgRNA positively regulates the reverse transcription process of pgRNA, promoting viral replication (Imam et al., 2018; Kim and Siddiqui, 2021; Lv et al., 2023).

m^6^A modification also exhibits a complex spatio-temporal specific regulatory pattern in SFTSV. SFTSV recruits the host methyltransferase complex METTL3-METTL14 via Np, prompting the translocation of this complex to the cytoplasm. By utilizing its C-lobe domain (P112-L245) to interact with the METTL3 zinc finger domain (ZFD) (aa 258-336), Np catalyzes the m^6^A modification of viral RNA (Chen et al., 2024). Simultaneously, Np synergistically recruits the RNA-binding protein family GF2BP1/2/3, which binds to m^6^A modification sites to significantly enhance the stability of viral genomic RNA and the translational efficiency of Np mRNA, thereby driving efficient viral replication (Chen et al., 2024). This is similar to the mechanism found in SARS-CoV-2 infection, where RdRp appears to regulate the synthesis and ubiquitination of METTL3 through unknown mechanisms, altering its distribution and enhancing viral replication (Li et al., 2021). This demonstrates that m^6^A modification plays a crucial role in viral replication.

Furthermore, the m^6^A reader protein YTHDF1 exhibits functional duality during SFTSV infection. On one hand, YTHDF1/2/3 proteins recognize the A273 site to promote viral translation efficiency (Liu et al., 2024). On the other hand, YTHDF1 recognizes the A63 and A343 sites to negatively regulate SFTSV mRNA translation efficiency; specifically, YTHDF1 reduces the RNA stability and protein translation efficiency of the SFTSV S segment in an m^6^A-dependent manner (Liu et al., 2024). To dismantle the negative regulatory effect of host YTHDF1, SFTSV has evolved a dual strategy. The first layer of this strategy is that, after SFTSV infection, total cellular lactate levels rise, promoting lysine lactylation (Klac) modification of YTHDF1, which leads to increased ubiquitination levels and decreased YTHDF1 stability. The second layer of the strategy involves the virulence factor NSs, which induces the nuclear translocation of the deacylase Sirt6 and interacts with it to downregulate the formation of the Sirt6-YTHDF1 complex. This weakens the removal of lysine lactylation (Klac) from the YTHDF1 protein by Sirt6, thereby affecting YTHDF1 protein stability and subsequently promoting viral replication (Liu et al., 2024). Consequently, SFTSV not only utilizes the host m^6^A writing and reading systems to enhance its own RNA stability and translation efficiency but also lifts host restrictions by regulating the post-translational modification status of m^6^A reader proteins, reflecting the virus’s sophisticated utilization of the host epitranscriptomic regulatory network (**Figure 3**).

**Figure 3:**
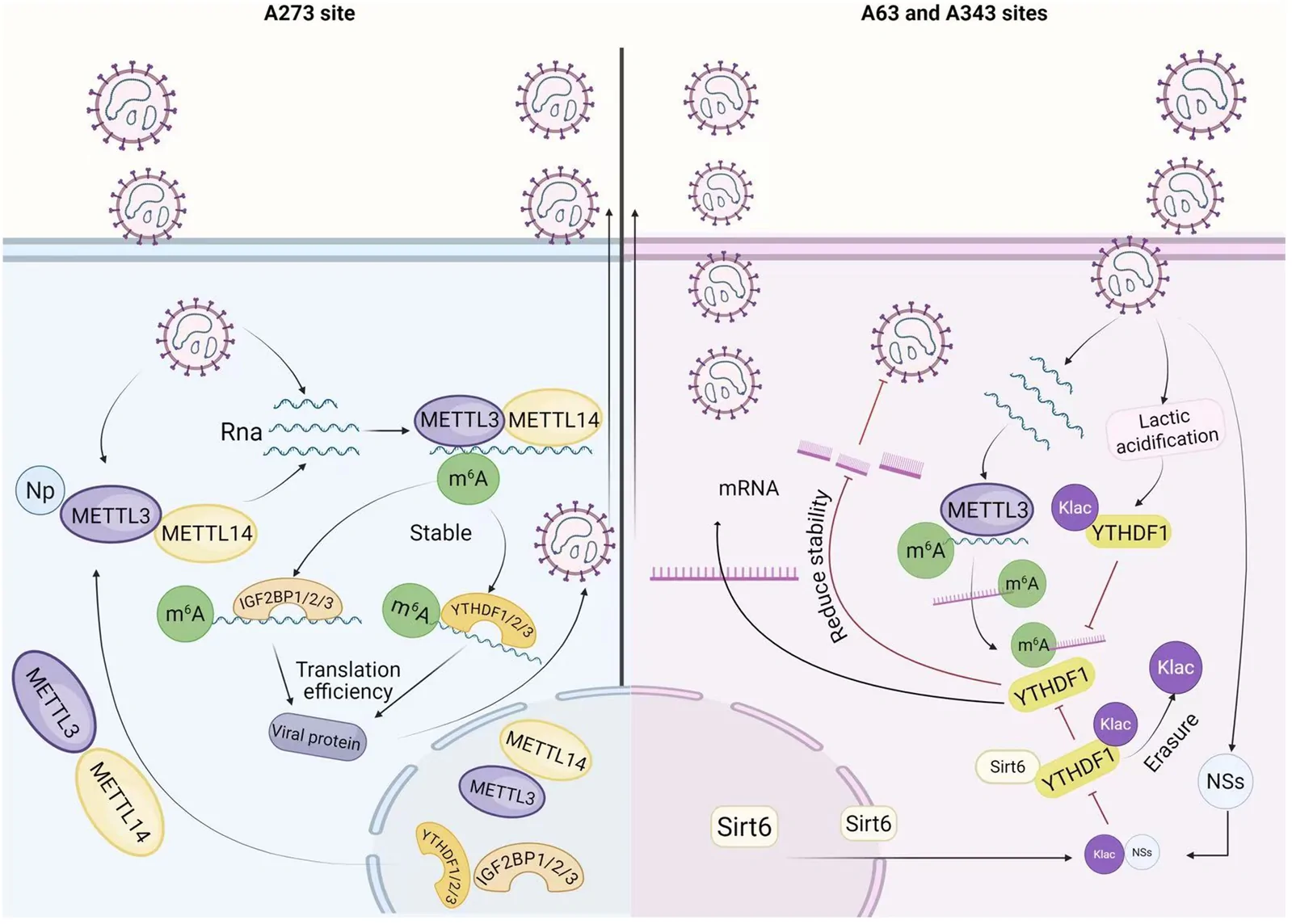
Bidirectional regulation of m^6^A modification in viral infection. This image compares the differential regulatory roles of SFTSV in host RNA epitranscriptomic modifications and viral replication in the context of mutational backgrounds at the A273 site versus the A63/A343 sites. The left panel illustrates that at the A273 site, the viral nucleocapsid protein (Np) interacts with the host methyltransferase complex METTL3/METTL14 to promote the m⁶A modification of viral RNAs. The m⁶A-modified viral RNAs are subsequently recognized and bound by reader proteins such as YTHDF1/2/3 and IGF2BP1/2/3. This process significantly enhances RNA stability and translational efficiency, thereby promoting viral protein synthesis and viral replication. The right panel reveals that at the A63 and A343 sites, viral infection induces lactic acidification and alters the lysine lactylation (Kla) levels of host proteins, which directly affects the modification status of YTHDF1 and its ability to recognize m⁶A-marked RNAs. Meanwhile, Sirt6 is involved in removing the lactylation marks, a regulatory process that is also modulated by the viral NSs protein. Collectively, these alterations decrease viral mRNA stability and suppress related post-transcriptional regulation. This suggests that variations at distinct sites can alter SFTSV replication efficiency and the pathogenic process by modulating m⁶A recognition, RNA fate, and host translational regulatory networks.

## 5 Programmed cell death and pathogenesis of SFTSV-infected host cells

By suppressing host antiviral responses, viruses create a microenvironment conducive to their own replication, completing gene transcription and translation and proceeding to the assembly and release of viral particles. Simultaneously, the host utilizes various programmed cell death (PCD) pathways to restrict infection and eliminate infected cells. PCD includes modes such as apoptosis, necroptosis, pyroptosis, autophagy-dependent cell death, and ferroptosis, which are not only involved in maintaining tissue homeostasis but also serve as key components of the innate immune defense system (Lamkanfi and Dixit, 2010). The interaction between the virus and the host leads to a complex bidirectional regulation of cell death. On one hand, the host limits viral dissemination and promotes tissue repair by inducing programmed death; conversely, viruses can selectively regulate or exploit specific death pathways to facilitate replication or evade immune clearance. Emerging research demonstrates that SFTSV possesses the capability to modulate host cell death through multiple pathways. At the inflammatory level, SFTSV can activate the NLRP3 inflammasome to drive the cleavage and maturation of caspase-1, promoting the release of IL-1β and IL-18 (Liu et al., 2021; Li et al., 2022; Liu PP. et al., 2025). This pyroptotic mechanism shares pathological features with SARS-CoV-2 infection in monocytes and HIV-induced pyroptosis in CD4+ T cells (Chen et al., 2022; Lokupathirage et al., 2025). Beyond pyroptosis, SFTSV also participates in inflammatory pathogenesis via RIPK3-MLKL-mediated necroptosis (Li S. et al., 2025). The viral non-structural protein NSs directly binds to the kinase domain of RIPK3 and forms condensate-like “viral necrosomes,” which promote RIPK3 autophosphorylation and MLKL activation. By inducing inflammatory necroptosis while inhibiting RIPK3-mediated antiviral apoptosis in a kinase-independent manner, the virus amplifies the inflammatory response and facilitates its own replication (Li S. et al., 2025). This suggests that SFTSV may reshape RIPK3-downstream cell death programs via NSs, suppressing antiviral apoptosis while driving more pro-inflammatory necroptosis to achieve a pathogenic outcome where viral replication thrives alongside host damage. Regarding metabolic regulation, SFTSV utilizes m^6^A epigenetic modifications to regulate the ATG5-dependent ferritinophagy pathway, triggering hepatocyte ferroptosis and hepatic dysfunction (Liu B. et al., 2025); simultaneously, SFTSV coordinates pyroptosis, apoptosis, and autophagy to cause multifaceted damage to platelets, accelerating the clinical progression of thrombocytopenia (Ding et al., 2026). To date, the dynamic regulatory network of SFTSV-modulated host cell programmed death—particularly the synergistic or antagonistic interactions between various death modes within the infection microenvironment—remains a critical scientific challenge to be addressed.

Among the above-mentioned multiple programmed cell death pathways, the most systematically researched is the regulation of the viral life cycle by autophagy. Autophagy is a highly conserved intracellular metabolic regulatory mechanism, which transports intracellular damaged proteins, organelles, or pathogens to lysosomes for degradation through the formation of double-membrane enclosed vesicles, thereby achieving the recycling of cellular components and the maintenance of homeostasis (Zhou et al., 2025). The invasion of exogenous pathogens is the trigger for autophagy initiation. The interaction between viruses and cell surface receptors induces autophagy, such as inducing autophagy through the CD46-Cyt-1/GOPC pathway, TOLL-like receptors recognizing viruses and then stimulating adaptor proteins to interact with Beclin1 to induce autophagy, and integrins can activate autophagy by interacting with LIR motifs or inhibiting the mTOR pathway (Yang, 2023; Zhang J. et al., 2023). There are complex interactions between viruses and autophagy. As a protective mechanism of the body, it induces innate immune responses through antigen presentation and the induction of interferon production during viral infection, achieving the effect of clearing pathogens. However, in order to antagonize this effect, viruses have evolved multiple evasion strategies. Viruses disrupt the formation of autophagosomes through interference with various stages of autophagosome formation, can also hijack autophagosomes as sites for their own assembly and maturation, and complete the release of viral particles through autophagy. For example, SARS-CoV-2 inhibits autolysosomal degradation through NSP6, preventing recognition and degradation by the body’s defense system (Ivanova et al., 2023), and viruses such as HCV, ZIKV, and DENV have been confirmed to be able to induce autophagy, utilizing autophagy to promote their own replication (Ming et al., 2016). Therefore, depending on the differences in viruses and viral replication cycles, autophagy has both the function of promoting viral replication and anti-viral function.

SFTSV controls the viral life cycle by regulating autophagic flux. SFTSV assembles progeny SFTSV particles through phagocytic vesicles formed by the endoplasmic reticulum-Golgi intermediate compartment (Chen et al., 2023). In this process, Np/NSs encoded by the S segment induce complete autophagic flux (i.e., the entire process from autophagosome formation to lysosomal degradation) to promote viral replication (Feng et al., 2023).

Research has demonstrated that Np antagonizes the innate immune response by inducing mitophagy. After SFTSV invades host cells, viral RNA is recognized by RIG-I-like receptors, subsequently RIG-I translocates to the mitochondrial outer membrane and interacts with MAVS, the latter of which transmits signals to downstream kinases TBK1 and the IKK complex. IRF3, IRF7, and NF-κB are further activated and translocated into the nucleus, leading to the induction of type I interferons (IFN-I), IFN-stimulated genes (ISGs), and pro-inflammatory cytokines, thereby exerting antiviral effects. Np causes the RLR downstream pathway to be unable to activate by degrading MAVS through autophagy, thus antagonizing the antiviral response. Np induces TUFM (Tu translation elongation factor, mitochondrial)-dependent mitophagy, upregulates the expression of TUFM and interacts with it to translocate to the mitochondria, then mediates mitochondrial sequestration into autophagosomes through interaction with LC3, thereby inducing mitophagy for the degradation of MAVS (Zhang WK. et al., 2025). In addition, Np can also inhibit the association between BECN1 and BCL2, inhibiting its binding with BCL2 to promote PIK3C3-BECN1 complex-dependent autophagy (Wang T. et al., 2022).

In addition to Np, NSs utilizes autophagy to promote viral replication. NSs interacts with vimentin and induces the degradation of vimentin through the K48-linked ubiquitin-proteasome pathway, which hinders the formation of the negatively regulatory Beclin1-vimentin complex and promotes complete autophagic flux, thereby facilitating viral replication and release (Liu et al., 2023). Np and NSs not only regulate autophagy through single proteins, but can also interact with each other to jointly participate in the regulation of autophagy. Research has found that NSs W61Y (substitution of tryptophan with tyrosine at position 61) can inhibit the interaction between Np and NSs, thereby affecting the lipidation process of LC3-I, inhibiting the formation of phagophores, and ultimately affecting replication; moreover, viral components such as Np and NSs can also promote autophagy by inhibiting the phosphorylation of MTOR and suppressing the activation of its downstream pathways (Park et al., 2024) (**Figure 2**).

## 6 Interaction between SFTSV and platelets

### 6.1 Role of platelets in SFTSV infection

Platelets, as an important component of the blood system, play an important role in thrombosis and hemostasis. At the same time, platelets also play an important role in immune responses; their surfaces possess a variety of receptors related to immune functions, such as Toll-like receptors, NOD-like receptors, and the major histocompatibility complex, etc., and these surface receptors and proteins interact with invading pathogens to exert innate immune functions (Wolny et al., 2023; Sun et al., 2025b). Activated platelets release various chemokines and cytokines, participating in the regulation of inflammatory and immune responses (Tian et al., 2025).

In SFTSV infection, platelets, with their unique identity, become the key link connecting the virus and the host’s immune response. Activated platelets can regulate the phagocytic function of phagocytes and promote the release of multiple cytokines such as IL-6, IL-10, and TNF-α; while clearing the virus, this can lead to a cytokine storm and exacerbate disease severity (Liu et al., 2017). SFTSV adheres to platelets, utilizing platelets as carriers to enter phagocytes for replication, which can lead to the overactivation of phagocytes and the release of large amounts of inflammatory factors and chemokines (Li, 2013; Nakano et al., 2017; Du et al., 2024). Clinical studies show that the levels of inflammatory factors and chemokines in SFTS patients are significantly higher than those in healthy controls, and are often accompanied by lymphopenia and the inhibition of adaptive immune responses (He et al., 2021; Hou et al., 2025). In SARS virus infection, research has shown that T cell numbers are negatively correlated with serum IL-6, IL-10, and TNF-α; combined with the fact that the same phenomenon of the aforementioned cytokine elevation and T cell reduction exists in SFTSV infection (Li et al., 2023; Chen et al., 2025), it suggests that SFTSV may also partially through inflammatory factor imbalance indirectly inhibit T cell function, promote T cell exhaustion, ultimately weaken host antiviral immunity and drive the formation of cytokine storms, diminish the host’s ability to respond to the virus, seriously damage host immune function, cause the occurrence of host immunodeficiency diseases, increase the occurrence of opportunistic infections, and exacerbate the progression of the disease.

Beyond their role in mediating inflammatory amplification and viral transport, platelets are also direct targets of SFTSV damage, leading to functional impairment and a reduction in counts. SFTSV-induced platelet activation and apoptosis are the primary causes of thrombocytopenia, the severity of which correlates with that of SFTS. Recent studies indicate that platelet surface glycoproteins recognize and interact with SFTSV glycoproteins GN/GC through hydrogen bonds and electrostatic interactions, activating the GPVI signaling pathway and triggering downstream spleen tyrosine kinase (Syk), signal transducer and activator of transcription 3 (STAT3), and phospholipase Cγ2 (PLCγ2) (Fang et al., 2023). This series of reactions enhances the expression of P-selectin on the platelet surface and the exposure of phosphatidylserine (PS), causing platelets to be overactivated and aggregate at endothelial cells, increasing their risk of being cleared and the possibility of thrombosis. At the same time, the activated platelets release various chemokines, which not only may initiate immune responses and enhance phagocytosis, but also affect functions such as platelet aggregation, apoptosis, ATP release, spreading, and clot retraction, collectively promoting the loss and reduction in the number of platelets during SFTSV infection (Fang et al., 2025).

In parallel, SFTSV can internalize into platelets, exploiting their active metabolism to replicate within organelles such as mitochondria, the Golgi apparatus, and the endoplasmic reticulum (Cox, 2023). Platelets with viral adhesion or internalization are more likely to become targets for phagocytes. Transcriptomic analysis of SFTSV infection reveals that the virus can downregulate the expression of calcium signaling pathway-related genes GNA13 and ARHGEF12, thereby modulating genes such as ROCK1 and MYL12A to inhibit the calcium signaling pathway (Fu et al., 2023). Calcium signaling transduction plays a central role in platelet activation, granule release, adhesion, aggregation, and cytoskeletal remodeling; its dysregulation directly leads to abnormalities in platelet morphology and function (Mojzisch and Brehm, 2021). Newly released virions then interact with uninfected platelets, further exacerbating the depletion of platelets and adding a new dimension of complexity to the pathological mechanisms of SFTSV infection.

Concurrently, the interaction between SFTSV and platelets can induce a pronounced oxidative stress response. The adhesion of SFTSV triggers the generation of large amounts of reactive oxygen species (ROS) within platelets; an excess of ROS disrupts the platelet redox balance and, by upregulating Bax/Bak proteins, decreasing mitochondrial membrane potential, and activating MAPK pathways (p38, ERK, and JNK), promotes the release of cytochrome C and its binding with Apaf-1 (Zhou and Yu, 2021; Newman and Shadel, 2023; Zhou et al., 2024; Liao et al., 2025). This, in turn, activates the mitochondrial caspase apoptosis pathway, ultimately inducing platelet apoptosis. In this process, the activation of MAPK is a pivotal link; it not only enhances the activation state of platelets but also promotes platelet aggregation and clot retraction. Antioxidants exhibit a significant inhibitory effect on the elevated phosphorylation levels of MAPK family members (including ERK, p38, and JNK) caused by SFTSV infection, confirming that SFTSV induces apoptosis by utilizing the ROS-MAPK signaling pathway (Zhou et al., 2024).

In addition to oxidative stress, SFTSV infection can also induce extensive changes in the transcriptomic and functional states of platelets. Particularly notable among these are the mediation of neutrophil activation, type I interferon (IFN) signaling, and the fine regulation of the viral life cycle. In this series of complex biological responses, three differentially expressed genes (DEGs)—*LYZ* (lysozyme C), *USP1* (ubiquitin carboxyl-terminal hydrolase 1), and *CTSS* (cathepsin S)—play crucial roles, suggesting their potential involvement in the aforementioned processes (Fang et al., 2025). Although the specific roles of these three genes in SFTSV infection are not yet fully elucidated, existing studies have shown that they possess potential functions in other viral infections or immune regulation. Among them, USP1 can stabilize cGAS and enhance type I interferon responses through the UAF1-USP1 deubiquitinase complex, thereby promoting host antiviral immunity, which suggests that USP1 may play an important immunoregulatory role in SFTSV infection-related platelet responses (Yu et al., 2017). LYZ is an antimicrobial enzyme involved in innate immunity that primarily exerts antibacterial effects; in the context of porcine epidemic diarrhea virus (PEDV), it was found that LYZ downregulates the transcriptional level of RIG-I via YTHDF2, thereby affecting the degradation of endogenous RIG-I protein and inhibiting innate immunity (Zhang Y. et al., 2025). Meanwhile, CTSS is associated with neuroinflammation caused by human herpesvirus 6 (HHV-6) and multiple sclerosis (Shao et al., 2016). However, the specific functions of these three genes in SFTSV infection require further exploration. In addition, SFTSV infection leads to platelet activation and functional abnormalities, where activated platelets express a variety of receptors on their membrane surfaces that participate in platelet adhesion, aggregation, and the release of bioactive substances (such as growth factors, chemokines, and cytokines), playing important roles in thrombosis, hemostasis, and immunomodulation. For instance, the most abundant integrin on the platelet surface, αIIbβ3, binds to multiple ligands (e.g., fibrinogen, von Willebrand factor, and fibronectin) to activate integrin signaling involved in mediating platelet adhesion and aggregation to form thrombi (Son et al., 2017); GPVI is a key collagen receptor on the platelet surface that can also interact with fibrin and fibrinogen, whose activation can further mediate the activation of adhesion receptors such as integrins αIIbβ3 and α2β1, inducing downstream signal transduction and platelet granule release, thereby promoting thrombosis (Li R. et al., 2025); platelet P-selectin (CD62P) is stored in platelet α-granules and is exposed on the cell surface upon platelet activation through degranulation, and its interaction with the P-selectin glycoprotein ligand-1 (PSGL-1) on the neutrophil surface mediates the formation of neutrophil extracellular traps (Bautista-Becerril et al., 2021); furthermore, platelets can release extracellular vesicles expressing molecules such as GPIIb/IIIa, GPIbα, and P-selectin to participate in inflammatory regulation and angiogenesis. SFTSV typically results in reduced platelet membrane protein trafficking and impaired intracellular vesicle-related structures and transport functions, while platelet-derived vesicles themselves carry a procoagulant phenotype and participate in thrombus maintenance, inflammatory amplification, and angiogenesis; therefore, these abnormalities in lethal SFTS patients may hinder the transport and release of cytokines and bioactive molecules, thereby leading to the suppression of platelet activation and correlating with poor outcomes (Yan et al., 2023). PLA2G4A, as a key enzyme in the arachidonic acid metabolic pathway, mediates the release of arachidonic acid (AA) from membrane phospholipids; AA is further metabolized to produce bioactive thromboxane A2 (TXA2), and TXA2 is an important mediator for promoting platelet activation (Fu et al., 2023). SFTSV infection downregulates PI3-AKT pathway-related genes (GP1BA, PIK3CA, PIK3CB, AKT3, PRKG1, PRKG2), inhibiting the activation of PLA2G4A, thus suppressing TXA2 production and inhibiting platelet activation (Fu et al., 2023). RAP1 is an important intersection in platelet signaling pathways, which can activate integrin αIIbβ3 through Talin1 to mediate platelet attachment and adhesion (Lagarrigue et al., 2020). In patients infected with SFTSV, the activation of RAP1A, ACTB, and VAMP8 is suppressed, thereby inhibiting RAP1 signaling activation and subsequently affecting platelet attachment and adhesion (Fu et al., 2023; Shi et al., 2025). Histone methylation levels influence the outcomes of lethal SFTS patients (Fang et al., 2025). Relevant studies have shown that the reduction of histone trimethylation increases the binding of von Willebrand factor (vWF) to NF-κB and drives its transcription, enhancing platelet adhesion (Ghani and Pepke-Zaba, 2023). Increased histone methylation in lethal patients may disrupt this balance. Although platelets are anucleate cells, they possess a variety of functional coding or non-coding RNAs and translational mechanisms inherited from megakaryocyte precursors that have important biological significance, participating in platelet aggregation and adhesion and serving as one of the sources for synthesizing proteins adapted to their functions (Noh, 2021); as histone methylation is involved in regulating gene expression, alternative splicing, and the accessibility of transcription factors or regulatory complexes (Xu et al., 2024), an increase in histone methylation may also lead to platelet dysfunction, potentially correlating with disease severity (**Figure 4**).

**Figure 4:**
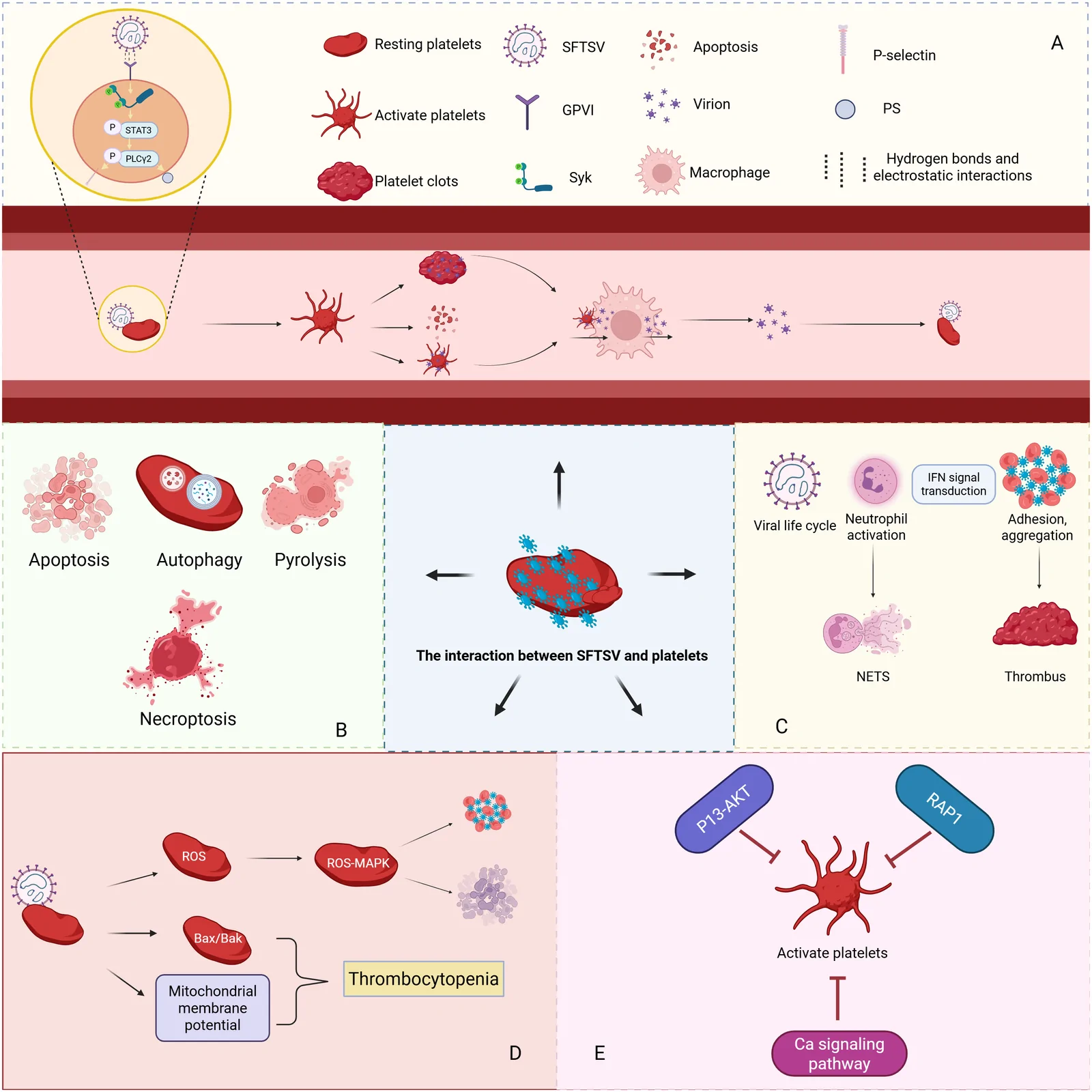
SFTSV interaction with platelets. **(A)** Interaction between SFTSV Gn/Gc and Circulating Platelets. **(B)** Platelet Death Pathway. **(C)** SFTSV Infection Alters Platelet Function, Primarily by Regulating the Viral Life Cycle, Mediating Neutrophil Activation, Participating in Type I Interferon Release, and Influencing Platelet Aggregation, Adhesion, and Thrombus Formation. **(D)** SFTSV induces platelet activation and apoptosis via the ROS-MAPK pathway, simultaneously upregulating Bax/Bak expression and altering platelet membrane potential, leading to thrombocytopenia. **(E)** SFTSV interacts with platelets to downregulate transcription levels of genes associated with P13- AKT, RAP1, and Ca signaling pathways, thereby inhibiting platelet activation.

## 7 Conclusion

This review summarizes the multidimensional mechanisms of the interaction between SFTSV and the host, delving into the complex network across multiple distinct levels, including viral protein functions, m^6^A modification, tick salivary factors, and platelet regulation. Viral proteins, as key effectors of SFTSV pathogenesis, not only directly participate in the viral life cycle but also influence disease progression by modulating host cell signaling pathways. As an emerging epigenetic regulatory mechanism, m^6^A modification has been found to affect SFTSV gene expression and virus-host interactions, playing a bidirectional regulatory role during infection and providing a new perspective for understanding viral pathogenic mechanisms. Furthermore, as ticks serve as the primary transmission vector for SFTSV, HIDfsin2 in their saliva plays an important role in viral transmission and host immune responses. Moreover, platelets, as a core component of the host immune and coagulation systems, are both targets of viral attack and participants in antiviral defense during SFTSV infection, and the elucidation of their regulatory mechanisms provides potential targets for the development of therapeutic strategies. In summary, the interaction between SFTSV and the host is a complex process involving multiple factors and levels; future research needs to further explore the fine regulatory networks of these mechanisms, laying a solid foundation for the development of targeted therapeutic drugs and preventive vaccines.

## References

[B1] Ahmad F. Kannan M. Ansari A. W. (2022). Role of SARS-CoV-2 -induced cytokines and growth factors in coagulopathy and thromboembolism. Cytokine Growth Factor Rev. 63, 58–68. doi: 10.1016/j.cytogfr.2021.10.007 34750061 PMC8541834

[B2] Amini-Bavil-Olyaee S. Choi Y. J. Lee J. H. Shi M. Huang I. C. Farzan M. . (2013). The antiviral effector IFITM3 disrupts intracellular cholesterol homeostasis to block viral entry. Cell Host Microbe 13, 452–464. doi: 10.1016/j.chom.2013.03.006 23601107 PMC3646482

[B3] Amurri L. Horvat B. Iampietro M. (2023). Interplay between RNA viruses and cGAS/STING axis in innate immunity. Front. Cell. Infect. Microbiol. 13, 1172739. doi: 10.3389/fcimb.2023.1172739 37077526 PMC10106766

[B4] Bartíková P. Kazimírová M. Štibrániová I. (2020). Ticks and the effects of their saliva on growth factors involved in skin wound healing. J. Venom Res. 10, 45–52. PMC7659473 33209253

[B5] Bautista-Becerril B. Campi-Caballero R. Sevilla-Fuentes S. Hernández-Regino L. M. Hanono A. Flores-Bustamante A. . (2021). Immunothrombosis in COVID-19: Implications of neutrophil extracellular traps. Biomolecules 11 (5), 694. doi: 10.3390/biom11050694 34066385 PMC8148218

[B6] Beaufays J. Adam B. Decrem Y. Prévôt P. P. Santini S. Brasseur R. . (2008). Ixodes ricinus tick lipocalins: identification, cloning, phylogenetic analysis and biochemical characterization. PloS One 3, e3941. doi: 10.1371/journal.pone.0003941 19096708 PMC2601031

[B7] Ben Abdallah H. Marino G. Idorn M. L S. R. Bregnhøj A. Paludan S. R. . (2024). The heat shock protein 90 inhibitor RGRN-305 attenuates SARS-CoV-2 spike protein-induced inflammation *in vitro* but lacks effectiveness as COVID-19 treatment in mice. PloS One 19, e0310915. doi: 10.1371/journal.pone.0310915 39325762 PMC11426470

[B8] Bocos-Asenjo I. T. Niño-Sánchez J. Ginésy M. Diez J. J. (2022). New insights on the integrated management of plant diseases by RNA strategies: Mycoviruses and RNA interference. Int. J. Mol. Sci. 23 (16), 9236. doi: 10.3390/ijms23169236 36012499 PMC9409477

[B9] Cao Y. Ren Q. Chang S. Cui W. Zhao P. Wang Y. (2024). N6-methyladenosine RNA methylation modification regulates the transcription of viral-derived E (XSR) miRNAs to promote ALV-J replication. Vet. Microbiol. 298, 110218. doi: 10.1016/j.vetmic.2024.110218 39159504

[B10] Carvalho-Costa T. M. Mendes M. T. da Silva M. V. da Costa T. A. Tiburcio M. G. Anhê A. C. . (2015). Immunosuppressive effects of Amblyomma cajennense tick saliva on murine bone marrow-derived dendritic cells. Parasit Vectors 8, 22. doi: 10.1186/s13071-015-0634-7 25586117 PMC4304185

[B11] Chable-Bessia C. Meziane O. Latreille D. Triboulet R. Zamborlini A. Wagschal A. . (2009). Suppression of HIV-1 replication by microRNA effectors. Retrovirology 6, 26. doi: 10.1186/1742-4690-6-26 19272132 PMC2657893

[B12] Chang M. Min Y. Q. Xu Z. Deng F. Wang H. Ning Y. J. (2024). Host factor MxA restricts Dabie bandavirus infection by targeting the viral NP protein to inhibit NP-RdRp interaction and ribonucleoprotein activity. J. Virol. 98, e0156823. doi: 10.1128/jvi.01568-23 38054738 PMC10805036

[B13] Chen L. Chen T. Li R. Xu Y. Xiong Y. (2023). Recent advances in the study of the immune escape mechanism of SFTSV and its therapeutic agents. Viruses 15 (4), 940. doi: 10.3390/v15040940 37112920 PMC10142331

[B14] Chen X. Zhang W. Yi W. Yang L. Bi X. Lin Y. . (2022). Pathway of cell death and its role in virus infection. Viral Immunol. doi: 10.1089/vim.2022.0010 35758840

[B15] Chen Y. Miller H. Benlagha K. Byazrova M. G. Filatov A. V. Zhang Z. . (2025). A focus on the mechanisms of alteration in host lymphocyte level following severe fever with thrombocytopenia syndrome virus (SFTSV) infection. J. Inflamm. Res. 18, 13265–13277. doi: 10.2147/jir.S531068 41031180 PMC12477067

[B16] Chen Z. Zhang J. Wang J. Tong H. Pan W. Ma F. . (2024). N6-methyladenosine RNA modification promotes severe fever with thrombocytopenia syndrome virus infection. PloS Pathog. 20, e1012725. doi: 10.1371/journal.ppat.1012725 39585899 PMC11627400

[B17] Cheng M. Zhang R. Li J. Ma W. Li L. Jiang N. . (2024). MβCD inhibits SFTSV entry by disrupting lipid raft structure of the host cells. Antiviral Res. 231, 106004. doi: 10.1016/j.antiviral.2024.106004 39265655

[B18] Cholvi M. Trelis M. Bueno-Marí R. Khoubbane M. Gil R. Marcilla A. . (2024). Wolbachia infection through hybridization to enhance an incompatible insect technique-based suppression of Aedes albopictus in Eastern Spain. Insects 15 (3), 206. doi: 10.3390/insects15030206 38535401 PMC10971076

[B19] Courtney D. G. Kennedy E. M. Dumm R. E. Bogerd H. P. Tsai K. Heaton N. S. . (2017). Epitranscriptomic enhancement of influenza A virus gene expression and replication. Cell Host Microbe 22, 377–386.e5. doi: 10.1016/j.chom.2017.08.004 28910636 PMC5615858

[B20] Cox D. (2023). Sepsis - it is all about the platelets. Front. Immunol. 14, 1210219. doi: 10.3389/fimmu.2023.1210219 37350961 PMC10282552

[B21] Dai W. Liu F. Li C. Lu Y. Lu X. Du S. . (2016). Blockade of Wnt/β-catenin pathway aggravated silica-induced lung inflammation through tregs regulation on Th immune responses. Mediators Inflamm., 6235614. doi: 10.1155/2016/6235614 27069316 PMC4812397

[B22] Ding Y. Hu Q. Hu Y. Yang Y. Chen J. Zhao F. . (2026). Immunopathogenesis of severe fever with thrombocytopenia syndrome: Core driving role of cytokine storm. Curr. Issues Mol. Biol. 48 (3), 263. doi: 10.3390/cimb48030263 41899415 PMC13025814

[B23] Du Q. Yu J. Chen Q. Chen X. Jiang Q. Deng L. . (2024). Clinical characteristics and influencing factors of severe fever with thrombocytopenia syndrome complicated by viral myocarditis: a retrospective study. BMC Infect. Dis. 24, 240. doi: 10.1186/s12879-024-09096-4 38389047 PMC10885462

[B24] Du S. Peng R. Xu W. Qu X. Wang Y. Wang J. . (2023). Cryo-EM structure of severe fever with thrombocytopenia syndrome virus. Nat. Commun. 14, 6333. doi: 10.1038/s41467-023-41804-7 37816705 PMC10564799

[B25] Esteves E. Obellianne C. Thangamani S. Hermance M. E. (2025). Protocol for encapsulating ticks to study tick hematophagy and tick-virus-host interactions. STAR Protoc. 6, 103925. doi: 10.1016/j.xpro.2025.103925 40591457 PMC12268535

[B26] Fang Y. Shen S. Zhang J. Xu L. Wang T. Fan L. . (2025). Thrombocytopenia in severe fever with thrombocytopenia syndrome due to platelets with altered function undergoing cell death pathways. J. Infect. Dis. 231, e183–e194. doi: 10.1093/infdis/jiae355 38996045 PMC11793052

[B27] Fang L. Yu S. Tian X. Fu W. Su L. Chen Z. . (2023). Severe fever with thrombocytopenia syndrome virus replicates in platelets and enhances platelet activation. J. Thromb. Haemost 21, 1336–1351. doi: 10.1016/j.jtha.2023.02.006 36792011

[B28] Feng K. Zhang H. Jiang Z. Zhou M. Min Y. Q. Deng F. . (2023). SFTS bunyavirus NSs protein sequestrates mTOR into inclusion bodies and deregulates mTOR-ULK1 signaling, provoking pro-viral autophagy. J. Med. Virol. 95, e28371. doi: 10.1002/jmv.28371 36458534

[B29] Fu H. Wang Y. Yuan C. Zhang Y. Zheng A. Zou Z. . (2023). Screening core genes and signaling pathways after SFTSV infection by integrated transcriptome profiling analysis. Virus Res. 332, 199138. doi: 10.1016/j.virusres.2023.199138 37211158 PMC10345746

[B30] Furuse Y. (2021). RNA modifications in genomic RNA of influenza A virus and the relationship between RNA modifications and viral infection. Int. J. Mol. Sci. 22 (17), 9127. doi: 10.3390/ijms22179127 34502037 PMC8431438

[B31] Ghani H. Pepke-Zaba J. (2023). Chronic thromboembolic pulmonary hypertension: A review of the multifaceted pathobiology. Biomedicines 12 (1), 46. doi: 10.3390/biomedicines12010046 38255153 PMC10813488

[B32] Gokhale N. S. McIntyre A. B. R. McFadden M. J. Roder A. E. Kennedy E. M. Gandara J. A. . (2016). N6-methyladenosine in Flaviviridae viral RNA genomes regulates infection. Cell Host Microbe 20, 654–665. doi: 10.1016/j.chom.2016.09.015 27773535 PMC5123813

[B33] Guimarães E. S. Marinho F. V. de Queiroz N. Antunes M. M. Oliveira S. C. (2021). Impact of STING inflammatory signaling during intracellular bacterial infections. Cells 11 (1), 74. doi: 10.3390/cells11010074 35011636 PMC8750390

[B34] Harsh S. Eleftherianos I. (2020). Flavivirus infection and regulation of host immune and tissue homeostasis in insects. Front. Immunol. 11, 618801. doi: 10.3389/fimmu.2020.618801 33329613 PMC7733989

[B35] He Z. Wang B. Li Y. Hu K. Yi Z. Ma H. . (2021). Changes in peripheral blood cytokines in patients with severe fever with thrombocytopenia syndrome. J. Med. Virol. 93, 4704–4713. doi: 10.1002/jmv.26877 33590892 PMC8360139

[B36] Hermance M. E. Thangamani S. (2020). Utilization of RNA in situ hybridization to understand the cellular localization of Powassan virus RNA at the tick-virus-host interface. Front. Cell. Infect. Microbiol. 10, 172. doi: 10.3389/fcimb.2020.00172 32411615 PMC7198705

[B37] Hicks P. Manzoni T. B. Westover J. B. Petch R. J. Roper B. Gowen B. B. . (2024). Safety, immunogenicity, and efficacy of a recombinant vesicular stomatitis virus vectored vaccine against severe fever with thrombocytopenia syndrome virus and Heartland bandavirus. Vaccines (Basel) 12 (12), 1403. doi: 10.3390/vaccines12121403 39772063 PMC11728676

[B38] Hou H. Chen R. Jiang Y. Wei W. Wang Y. Huang M. . (2025). Autoantibody profiles and prognostic significance in severe fever with thrombocytopenia syndrome (SFTS) patients. J. Med. Virol. 97, e70266. doi: 10.1002/jmv.70266 39995275

[B39] Huang X. B. Yu X. Y. Fan J. W. Kang W. B. Bao L. Wei Y. M. . (2026). SFTSV NSs sequesters the complex of SFTSV NP-SAFA to suppress SAFA mediated immune response. J. Med. Virol. 98, e70774. doi: 10.1002/jmv.70774 41439738

[B40] Huang X. B. Zhang Y. Fan J. W. Yu X. Y. Fang Y. L. Ren J. X. . (2025). hnRNPA2B1 recognizes RNA virus SFTSV infection through mitochondrial DNA. mBio 16, e0166825. doi: 10.1128/mbio.01668-25 40689679 PMC12345236

[B41] Imam H. Khan M. Gokhale N. S. McIntyre A. B. R. Kim G. W. Jang J. Y. . (2018). N6-methyladenosine modification of hepatitis B virus RNA differentially regulates the viral life cycle. Proc. Natl. Acad. Sci. U.S.A. 115, 8829–8834. doi: 10.1073/pnas.1808319115 30104368 PMC6126736

[B42] Ivanova T. Mariienko Y. Mehterov N. Kazakova M. Sbirkov Y. Todorova K. . (2023). Autophagy and SARS-CoV-2-old players in new games. Int. J. Mol. Sci. 24 (9), 7734. doi: 10.3390/ijms24097734 37175443 PMC10178552

[B43] Iwanami S. Kitagawa K. Ohashi H. Asai Y. Shionoya K. Saso W. . (2020). Should a viral genome stay in the host cell or leave? A quantitative dynamics study of how hepatitis C virus deals with this dilemma. PloS Biol. 18, e3000562. doi: 10.1371/journal.pbio.3000562 32730280 PMC7392214

[B44] Jansen van Vuren P. Tiemessen C. T. Paweska J. T. (2011). Anti-nucleocapsid protein immune responses counteract pathogenic effects of Rift Valley fever virus infection in mice. PloS One 6, e25027. doi: 10.1371/journal.pone.0025027 21949840 PMC3174991

[B45] Javaid N. Jang T. W. Fu Y. Choi Y. (2025). SFTSV NSs interacts with AGO2 to regulate the RNAi pathway for viral replication. J. Virol. 99, e0220524. doi: 10.1128/jvi.02205-24 40013801 PMC11998505

[B46] Jeon P. Yoo B. Kim Y. Lee S. Y. Woo H. M. Lim H. Y. . (2024). Characterization of high-affinity antibodies against the surface Gc protein of Dabie bandavirus / severe fever with thrombocytopenia syndrome virus. Biochem. Biophys. Rep. 39, 101779. doi: 10.1016/j.bbrep.2024.101779 39099605 PMC11296068

[B47] Jia Y. Li F. Liu Z. Liu S. Huang M. Gao X. . (2024). Interaction between the SFTSV envelope glycoprotein Gn and STING inhibits the formation of the STING-TBK1 complex and suppresses the NF-κB signaling pathway. J. Virol. 98, e0181523. doi: 10.1128/jvi.01815-23 38421179 PMC10949458

[B48] Jiang Z. Z. Chu M. Yan L. N. Zhang W. K. Li B. Xu J. . (2024). SFTSV nucleoprotein mediates DNA sensor cGAS degradation to suppress cGAS-dependent antiviral responses. Microbiol. Spectr 12, e0379623. doi: 10.1128/spectrum.03796-23 38712963 PMC11237745

[B49] Jiang X. M. Xin Q. L. Liu K. Peng X. F. Han S. Zhang L. Y. . (2023). Regulation of the WNT-CTNNB1 signaling pathway by severe fever with thrombocytopenia syndrome virus in a cap-snatching manner. mBio 14, e0168823. doi: 10.1128/mbio.01688-23 37882780 PMC10746258

[B50] Jin Z. Taguwa S. Hirano J. Uemura K. Ono C. Saito A. . (2025). SFTSV utilizes AXL/GAS6 for entry via PI3K-PLC-dependent macropinocytosis activated by AXL-kinase. J. Virol. 99, e0022125. doi: 10.1128/jvi.00221-25 40853130 PMC12455926

[B51] Kamal A. Boehm M. F. Burrows F. J. (2004). Therapeutic and diagnostic implications of Hsp90 activation. Trends Mol. Med. 10, 283–290. doi: 10.1016/j.molmed.2004.04.006 15177193 PMC7128344

[B52] Kim S. Jeon K. Choi H. Jeong D. E. Kang J. G. Cho N. H. (2024). Comparative analysis of the efficacy of vaccines using structural protein subunits of the severe fever with thrombocytopenia syndrome virus. Front. Microbiol. 15, 1348276. doi: 10.3389/fmicb.2024.1348276 38567080 PMC10985320

[B53] Kim D. Lai C. J. Cha I. Jung J. U. (2024). Current progress of severe fever with thrombocytopenia syndrome virus (SFTSV) vaccine development. Viruses 16 (1), 128. doi: 10.3390/v16010128 38257828 PMC10818334

[B54] Kim E. H. Park S. J. (2023). Emerging tick-borne Dabie bandavirus: Virology, epidemiology, and prevention. Microorganisms 11 (9), 2309. doi: 10.3390/microorganisms11092309 37764153 PMC10536723

[B55] Kim G. W. Siddiqui A. (2021). Hepatitis B virus X protein recruits methyltransferases to affect cotranscriptional N6-methyladenosine modification of viral/host RNAs. Proc. Natl. Acad. Sci. U.S.A. 118 (3), e2019455118. doi: 10.1073/pnas.2019455118 33397803 PMC7826408

[B56] Kubota M. Hashiguchi T. (2021). Unique tropism and entry mechanism of mumps virus. Viruses 13 (9), 1746. doi: 10.3390/v13091746 34578327 PMC8471308

[B57] Lagarrigue F. Paul D. S. Gingras A. R. Valadez A. J. Sun H. Lin J. . (2020). Talin-1 is the principal platelet Rap1 effector of integrin activation. Blood 136, 1180–1190. doi: 10.1182/blood.2020005348 32518959 PMC7472713

[B58] Lamkanfi M. Dixit V. M. (2010). Manipulation of host cell death pathways during microbial infections. Cell Host Microbe 8, 44–54. doi: 10.1016/j.chom.2010.06.007 20638641

[B59] Laureti M. Paradkar P. N. Fazakerley J. K. Rodriguez-Andres J. (2020). Superinfection exclusion in mosquitoes and its potential as an arbovirus control strategy. Viruses 12 (11), 1259. doi: 10.3390/v12111259 33167513 PMC7694488

[B60] Lee K. Seok J. H. Kim H. Park S. Lee S. Bae J. Y. . (2023). Genome-informed investigation of the molecular evolution and genetic reassortment of severe fever with thrombocytopenia syndrome virus. PloS Negl.Trop Dis. 17, e0011630. doi: 10.1371/journal.pntd.0011630 37713429 PMC10529592

[B61] Lee J. K. Shin O. S. (2021). Nonstructural protein of severe fever with thrombocytopenia syndrome phlebovirus inhibits TBK1 to evade interferon-mediated response. J. Microbiol. Biotechnol. 31, 226–232. doi: 10.4014/jmb.2008.08048 33397830 PMC9705905

[B62] Li D. (2013). A highly pathogenic new bunyavirus emerged in China. Emerg. Microbes Infect. 2, e1. doi: 10.1038/emi.2013.1 26038435 PMC3630492

[B63] Li M. (2024). Innate immune response against vector-borne bunyavirus infection and viral countermeasures. Front. Cell. Infect. Microbiol. 14, 1365221. doi: 10.3389/fcimb.2024.1365221 38711929 PMC11070517

[B64] Li S. Hao L. Zhang J. Deng J. Hu X. (2023). Focus on T cell exhaustion: new advances in traditional Chinese medicine in infection and cancer. Chin. Med. 18, 76. doi: 10.1186/s13020-023-00785-x 37355637 PMC10290330

[B65] Li Z. Hu J. Bao C. Gao C. Zhang N. Cardona C. J. . (2022). Activation of the NLRP3 inflammasome and elevation of interleukin-1β secretion in infection by sever fever with thrombocytopenia syndrome virus. Sci. Rep. 12, 2573. doi: 10.1038/s41598-022-06229-0 35173184 PMC8850576

[B66] Li N. Hui H. Bray B. Gonzalez G. M. Zeller M. Anderson K. G. . (2021). METTL3 regulates viral m^6^A RNA modification and host cell innate immune responses during SARS-CoV-2 infection. Cell Rep. 35, 109091. doi: 10.1016/j.celrep.2021.109091 33961823 PMC8090989

[B67] Li S. Li H. Zhou Z. Ye M. Wang Y. Li W. . (2025). A viral necrosome mediates direct RIPK3 activation to promote inflammatory necroptosis. Proc. Natl. Acad. Sci. U.S.A. 122, e2420245122. doi: 10.1073/pnas.2420245122 40424123 PMC12146775

[B68] Li R. Qiu Z. Cui Y. Xiang Q. (2025). Blocking platelet glycoprotein VI (GPVI) as a promising antithrombotic treatment. Drug Discov. Today 30, 104473. doi: 10.1016/j.drudis.2025.104473 40939697

[B69] Liao R. Wang L. Zeng J. Tang X. Huang M. Kantawong F. . (2025). Reactive oxygen species: Orchestrating the delicate dance of platelet life and death. Redox Biol. 80, 103489. doi: 10.1016/j.redox.2025.103489 39764976 PMC11759559

[B70] Lichinchi G. Gao S. Saletore Y. Gonzalez G. M. Bansal V. Wang Y. . (2016). Dynamics of the human and viral m(6)A RNA methylomes during HIV-1 infection of T cells. Nat. Microbiol. 1, 16011. doi: 10.1038/nmicrobiol.2016.11 27572442 PMC6053355

[B71] Liu J. W. Chu M. Jiao Y. J. Zhou C. M. Qi R. Yu X. J. (2021). SFTSV infection induced interleukin-1β secretion through NLRP3 inflammasome activation. Front. Immunol. 12, 595140. doi: 10.3389/fimmu.2021.595140 33708197 PMC7940371

[B72] Liu Q. He B. Huang S. Y. Wei F. Zhu X. Q. (2014). Severe fever with thrombocytopenia syndrome, an emerging tick-borne zoonosis. Lancet Infect. Dis. 14, 763–772. doi: 10.1016/s1473-3099(14)70718-2 24837566

[B73] Liu P. P. Jiang S. P. Li B. Gui W. T. Qin X. R. Yu X. J. (2025). The non-structural protein of SFTSV activates NLRP1 and CARD8 inflammasome through disrupting the DPP9-mediated ternary complex. PloS Pathog. 21, e1013258. doi: 10.1371/journal.ppat.1013258 40608794 PMC12225885

[B74] Liu M. M. Lei X. Y. Yu H. Zhang J. Z. Yu X. J. (2017). Correlation of cytokine level with the severity of severe fever with thrombocytopenia syndrome. Virol. J. 14, 6. doi: 10.1186/s12985-016-0677-1 28086978 PMC5237221

[B75] Liu S. Su Y. Lu Z. Zou X. Xu L. Teng Y. . (2023). The SFTSV nonstructural proteins induce autophagy to promote viral replication via interaction with vimentin. J. Virol. 97, e0030223. doi: 10.1128/jvi.00302-23 37039677 PMC10134822

[B76] Liu B. Tian X. Li L. Jiang N. Cheng M. Zhu J. . (2025). SFTSV induces liver ferroptosis through m^6^A-related ferritinophagy. Autophagy 21, 2353–2366. doi: 10.1080/15548627.2025.2503564 40340535 PMC12674464

[B77] Liu B. Tian X. Li L. Zhang R. Wu J. Jiang N. . (2024). Severe fever with thrombocytopenia syndrome virus induces lactylation of m^6^A reader protein YTHDF1 to facilitate viral replication. EMBO Rep. 25, 5599–5619. doi: 10.1038/s44319-024-00310-7 39496835 PMC11624280

[B78] Liu X. Xu Z. Tong Y. Wang C. Yao Y. Diao Y. . (2026). Investigation into the pancreatic pathogenesis of SFTSV across multiple levels. Adv. Sci. (Weinh) 13, e15862. doi: 10.1002/advs.202515862 41090515 PMC12767103

[B79] Lokupathirage S. M. W. Muthusinghe D. S. Sarii R. S. Akanbi O. A. Shimizu K. Tsuda Y. . (2025). Characterization of quasispecies of severe fever with thrombocytopenia syndrome virus. J. Virol. 99, e0179424. doi: 10.1128/jvi.01794-24 40202315 PMC12090785

[B80] Lv Z. Ran R. Yang Y. Xiang M. Su H. Huang J. (2023). The interplay between N6-methyladenosine and precancerous liver disease: Molecular functions and mechanisms. Discov. Oncol. 14, 78. doi: 10.1007/s12672-023-00695-2 37227534 PMC10212880

[B81] Ma S. Yan J. Barr T. Zhang J. Chen Z. Wang L. S. . (2021). The RNA m^6^A reader YTHDF2 controls NK cell antitumor and antiviral immunity. J. Exp. Med. 218 (8), e20210279. doi: 10.1084/jem.20210279 34160549 PMC8225680

[B82] Ming G. L. Tang H. Song H. (2016). Advances in Zika virus research: Stem cell models, challenges, and opportunities. Cell Stem Cell 19, 690–702. doi: 10.1016/j.stem.2016.11.014 27912090 PMC5218815

[B83] Mojzisch A. Brehm M. A. (2021). The manifold cellular functions of von Willebrand factor. Cells 10 (9), 2351. doi: 10.3390/cells10092351 34572000 PMC8466076

[B84] Moming A. Shi S. Shen S. Qiao J. Yue X. Wang B. . (2021). Fine mapping epitope on glycoprotein-Gn from severe fever with thrombocytopenia syndrome virus. PloS One 16, e0248005. doi: 10.1371/journal.pone.0248005 33651850 PMC7924767

[B85] Nakano A. Ogawa H. Nakanishi Y. Fujita H. Mahara F. Shiogama K. . (2017). Hemophagocytic lymphohistiocytosis in a fatal case of severe fever with thrombocytopenia syndrome. Intern. Med. 56, 1597–1602. doi: 10.2169/internalmedicine.56.6904 28626191 PMC5505921

[B86] Newman L. E. Shadel G. S. (2023). Mitochondrial DNA release in innate immune signaling. Annu. Rev. Biochem. 92, 299–332. doi: 10.1146/annurev-biochem-032620-104401 37001140 PMC11058562

[B87] Ning Y. J. Mo Q. Feng K. Min Y. Q. Li M. Hou D. . (2019). Interferon-γ-directed inhibition of a novel high-pathogenic phlebovirus and viral antagonism of the antiviral signaling by targeting STAT1. Front. Immunol. 10, 1182. doi: 10.3389/fimmu.2019.01182 31191546 PMC6546826

[B88] Noh J. Y. (2021). Megakaryopoiesis and platelet biology: Roles of transcription factors and emerging clinical implications. Int. J. Mol. Sci. 22 (17), 9615. doi: 10.3390/ijms22179615 34502524 PMC8431765

[B89] Park S. Kim C. (2026). Immune cells at the frontline of SFTSV infection. J. Microbiol. Biotechnol. 36, e2511002. doi: 10.4014/jmb.2511.11002 41742453 PMC12975498

[B90] Park E. M. Kim S. Lim S. Rahimizadeh P. Jeon H. Lim H. J. . (2025). Development of antibodies against severe fever with thrombocytopenia syndrome virus nucleoprotein for diagnosis. Appl. Microbiol. Biotechnol. 109, 139. doi: 10.1007/s00253-025-13530-1 40481890 PMC12145327

[B91] Park J. Y. Senevirathne A. Lloren K. K. S. Lee J. H. (2024). The effect of tryptophan-to-tyrosine mutation at position 61 of the nonstructural protein of severe fever with thrombocytopenia syndrome virus on viral replication through autophagosome modulation. Int. J. Mol. Sci. 25 (12), 6394. doi: 10.3390/ijms25126394 38928101 PMC11203599

[B92] Perumalsamy N. Sharma R. Subramanian M. Nagarajan S. A. (2024). Hard ticks as vectors: The emerging threat of tick-borne diseases in India. Pathogens 13 (7), 556. doi: 10.3390/pathogens13070556 39057783 PMC11279560

[B93] Pham M. Underwood J. Oliva Chávez A. S. (2021). Changing the recipe: Pathogen directed changes in tick saliva components. Int. J. Environ. Res. Public Health 18 (4), 1806. doi: 10.3390/ijerph18041806 33673273 PMC7918122

[B94] Ren X. Sun J. Kuang W. Yu F. Wang B. Wang Y. . (2024). A broadly protective antibody targeting glycoprotein Gn inhibits severe fever with thrombocytopenia syndrome virus infection. Nat. Commun. 15, 7009. doi: 10.1038/s41467-024-51108-z 39147753 PMC11327358

[B95] Schneider C. A. Calvo E. Peterson K. E. (2021). Arboviruses: How saliva impacts the journey from vector to host. Int. J. Mol. Sci. 22 (17), 9173. doi: 10.3390/ijms22179173 34502092 PMC8431069

[B96] Shao Q. Lin Z. Wu X. Tang J. Lu S. Feng D. . (2016). Transcriptome sequencing of neurologic diseases associated genes in HHV-6A infected human astrocyte. Oncotarget 7, 48070–48080. doi: 10.18632/oncotarget.10127 27344170 PMC5217001

[B97] Shen S. Zhang L. S. (2023). The regulation of antiviral innate immunity through non-m(6)A RNA modifications. Front. Immunol. 14, 1286820. doi: 10.3389/fimmu.2023.1286820 37915585 PMC10616867

[B98] Shen S. Zhang Y. Yin Z. Zhu Q. Zhang J. Wang T. . (2022). Antiviral activity and mechanism of the antifungal drug, anidulafungin, suggesting its potential to promote treatment of viral diseases. BMC Med. 20, 359. doi: 10.1186/s12916-022-02558-z 36266654 PMC9585728

[B99] Shi H. Chai P. Jia R. Fan X. (2020). Novel insight into the regulatory roles of diverse RNA modifications: Re-defining the bridge between transcription and translation. Mol. Cancer 19, 78. doi: 10.1186/s12943-020-01194-6 32303268 PMC7164178

[B100] Shi S. Gao J. Zhang Y. Zhan M. Tan Z. Wang P. . (2025). Inflammation and platelet hyperresponsiveness in coronary artery disease and the influence of Talin-1/αIIbβ3-mediated bidirectional signaling pathway. Front. Pharmacol. 16, 1535182. doi: 10.3389/fphar.2025.1535182 40183091 PMC11965607

[B101] Shrivastava G. Valenzuela-Leon P. C. Botello K. Calvo E. (2024). Aedes aEgypti saliva modulates inflammasome activation and facilitates flavivirus infection *in vitro*. iScience 27, 108620. doi: 10.1016/j.isci.2023.108620 38188518 PMC10770497

[B102] Šimo L. Kazimirova M. Richardson J. Bonnet S. I. (2017). The essential role of tick salivary glands and saliva in tick feeding and pathogen transmission. Front. Cell. Infect. Microbiol. 7, 281. doi: 10.3389/fcimb.2017.00281 28690983 PMC5479950

[B103] Son Y. M. Jeong D. H. Park H. J. Rhee M. H. (2017). The inhibitory activity of ginsenoside Rp4 in adenosine diphosphate-induced platelet aggregation. J. Ginseng Res. 41, 96–102. doi: 10.1016/j.jgr.2016.01.003 28123327 PMC5223082

[B104] Stamper A. Bisom T. Meade-White K. Lewis M. Leventhal S. Clancy C. . (2025). A replicating RNA vaccine protects against severe fever with thrombocytopenia syndrome virus infection in mice. NPJ Vaccines 10, 219. doi: 10.1038/s41541-025-01269-9 41102248 PMC12533269

[B105] Sun Z. Cheng J. Bai Y. Cao L. Xie D. Deng F. . (2023). Architecture of severe fever with thrombocytopenia syndrome virus. Protein Cell 14, 914–918. doi: 10.1093/procel/pwad019 37038326 PMC10691843

[B106] Sun H. Hu Q. Lu S. Yang Y. Zhang L. Long J. . (2025a). Current status of severe fever with thrombocytopenia syndrome in China (Review). Int. J. Mol. Med. 56 (5), 169. doi: 10.3892/ijmm.2025.5610 40849814 PMC12373438

[B107] Sun C. Shi H. Zhao X. Chang Y. L. Wang X. Zhu S. . (2023). The activation of cGAS-STING in acute kidney injury. J. Inflamm. Res. 16, 4461–4470. doi: 10.2147/jir.S423232 37842189 PMC10576462

[B108] Sun H. Zhu G. Li S. Li P. Zhang J. Yin R. . (2025b). Fucosylated glycosaminoglycan oligosaccharide HS14, derived from sea cucumbers, is a novel inhibitor of platelet toll-like receptor 2. Mar. Drugs 23 (3), 110. doi: 10.3390/md23030110 40137296 PMC11943722

[B109] Takahashi T. Suzuki T. (2011). Function of membrane rafts in viral lifecycles and host cellular response. Biochem. Res. Int. 2011, 245090. doi: 10.1155/2011/245090 22191032 PMC3235436

[B110] Tian Y. Zong Y. Pang Y. Zheng Z. Ma Y. Zhang C. . (2025). Platelets and diseases: signal transduction and advances in targeted therapy. Signal. Transduct Target Ther. 10, 159. doi: 10.1038/s41392-025-02198-8 40374650 PMC12081703

[B111] Tipih T. Burt F. J. (2020). Crimean-Congo hemorrhagic fever virus: Advances in vaccine development. Biores Open Access 9, 137–150. doi: 10.1089/biores.2019.0057 32461819 PMC7247048

[B112] Tirloni L. Kim T. K. Pinto A. F. M. Yates J. R. da Silva Vaz I. Jr. Mulenga A. . (2017). Tick-host range adaptation: Changes in protein profiles in unfed adult Ixodes scapularis and Amblyomma americanum saliva stimulated to feed on different hosts. Front. Cell. Infect. Microbiol. 7, 517. doi: 10.3389/fcimb.2017.00517 29312895 PMC5742094

[B113] Vogel D. Thorkelsson S. R. Quemin E. R. J. Meier K. Kouba T. Gogrefe N. . (2020). Structural and functional characterization of the severe fever with thrombocytopenia syndrome virus L protein. Nucleic Acids Res. 48, 5749–5765. doi: 10.1093/nar/gkaa253 32313945 PMC7261188

[B114] Wang L. Liu Y. Pang R. Guo Y. Ren Y. Wu Y. . (2024). The tick saliva peptide HIDfsin2 TLR4-dependently inhibits the tick-borne severe fever with thrombocytopenia syndrome virus in mouse macrophages. Antibiotics (Basel) 13 (5), 449. doi: 10.3390/antibiotics13050449 38786177 PMC11117380

[B115] Wang L. Sun F. Hu J. Zuo W. Zheng Y. Wu Y. . (2023). The tick saliva peptide HIDfsin2 promotes the tick-borne virus SFTSV replication *in vitro* by enhancing p38 signal pathway. Arch. Toxicol. 97, 1783–1794. doi: 10.1007/s00204-023-03515-2 37148319 PMC10163292

[B116] Wang B. Tang M. Yuan Z. Li Z. Hu B. Bai X. . (2022). Targeted delivery of a STING agonist to brain tumors using bioengineered protein nanoparticles for enhanced immunotherapy. Bioact Mater. 16, 232–248. doi: 10.1016/j.bioactmat.2022.02.026 35386310 PMC8965725

[B117] Wang Y. Wu H. Sun J. Kuang W. Wang H. Ning Y. J. . (2025). Structural insight into RNA encapsidation by the severe fever with thrombocytopenia syndrome virus nucleocapsid protein. mBio 16, e0238125. doi: 10.1128/mbio.02381-25 41170989 PMC12691673

[B118] Wang T. Xu L. Zhu B. Wang J. Zheng X. (2022). Immune escape mechanisms of severe fever with thrombocytopenia syndrome virus. Front. Immunol. 13, 937684. doi: 10.3389/fimmu.2022.937684 35967309 PMC9366518

[B119] Wang B. Zhang L. Deng F. Hu Z. Wang M. Liu J. (2024). Hsp90 β is critical for the infection of severe fever with thrombocytopenia syndrome virus. Virol. Sin. 39, 113–122. doi: 10.1016/j.virs.2023.11.008 38008382 PMC10877427

[B120] Williams G. D. Gokhale N. S. Horner S. M. (2019). Regulation of viral infection by the RNA modification N6-methyladenosine. Annu. Rev. Virol. 6, 235–253. doi: 10.1146/annurev-virology-092818-015559 31283446 PMC6884077

[B121] Williams H. M. Thorkelsson S. R. Vogel D. Busch C. Milewski M. Cusack S. . (2024). Structural snapshots of phenuivirus cap-snatching and transcription. Nucleic Acids Res. 52, 6049–6065. doi: 10.1093/nar/gkae330 38709882 PMC11162785

[B122] Williams H. M. Thorkelsson S. R. Vogel D. Milewski M. Busch C. Cusack S. . (2023). Structural insights into viral genome replication by the severe fever with thrombocytopenia syndrome virus L protein. Nucleic Acids Res. 51, 1424–1442. doi: 10.1093/nar/gkac1249 36651274 PMC9943659

[B123] Woldeamanuel G. G. Tlaye K. G. Wang X. Nguyen-Hoang L. Zhou Q. Wang Y. . (2025). Platelets in preeclampsia: An observational study of indices associated with aspirin nonresponsiveness, activation and transcriptional landscape. BMC Med. 23, 346. doi: 10.1186/s12916-025-04132-9 40490753 PMC12150514

[B124] Wolny M. Rozanova S. Knabbe C. Pfeiffer K. Barkovits K. Marcus K. . (2023). Changes in the proteome of platelets from patients with critical progression of COVID-19. Cells 12 (17), 2191. doi: 10.3390/cells12172191 37681923 PMC10486756

[B125] Xing C. Zhang C. Xu Z. Wang Y. Lu W. Liu X. . (2025). Genome-wide CRISPR screening identifies LRP1 as an entry factor for SFTSV. Nat. Commun. 16, 4036. doi: 10.1038/s41467-025-59305-0 40301361 PMC12041282

[B126] Xu B. Ye X. Wen Z. Chen S. Wang J. (2024). Epigenetic regulation of megakaryopoiesis and platelet formation. Haematologica 109, 3125–3137. doi: 10.3324/haematol.2023.284951 38867584 PMC11443398

[B127] Yan C. Wu H. Fang X. He J. Zhu F. (2023). Platelet, a key regulator of innate and adaptive immunity. Front. Med. (Lausanne) 10, 1074878. doi: 10.3389/fmed.2023.1074878 36968817 PMC10038213

[B128] Yanagi Y. Watanabe T. Hara Y. Sato Y. Kimura H. Murata T. (2022). EBV exploits RNA m(6)A modification to promote cell survival and progeny virus production during lytic cycle. Front. Microbiol. 13, 870816. doi: 10.3389/fmicb.2022.870816 35783391 PMC9240777

[B129] Yang J. (2023). Viruses binding to host receptors interacts with autophagy. Int. J. Mol. Sci. 24 (4), 3423. doi: 10.3390/ijms24043423 36834833 PMC9968160

[B130] Yang Y. Hsu P. J. Chen Y. S. Yang Y. G. (2018). Dynamic transcriptomic m(6)A decoration: Writers, erasers, readers and functions in RNA metabolism. Cell Res. 28, 616–624. doi: 10.1038/s41422-018-0040-8 29789545 PMC5993786

[B131] Yang W. Zhao Y. Yang Y. (2024). Dynamic RNA methylation modifications and their regulatory role in mammalian development and diseases. Sci. China Life Sci. 67, 2084–2104. doi: 10.1007/s11427-023-2526-2 38833084

[B132] Ying R. S. Zhu C. Fan X. G. Li N. Tian X. F. Liu H. B. . (2007). Hepatitis B virus is inhibited by RNA interference in cell culture and in mice. Antiviral Res. 73, 24–30. doi: 10.1016/j.antiviral.2006.05.022 16844238

[B133] Yu Y. Li J. Liu Q. Zhai R. Dai Y. Sun L. (2025). Advances in research on severe fever with thrombocytopenia syndrome virus. Front. Microbiol. 16, 1622394. doi: 10.3389/fmicb.2025.1622394 40771693 PMC12325320

[B134] Yu T. M. Li Z. M. Zhang W. K. Li B. Liu Q. Zhou C. M. . (2025). SFTSV NSs degrades SAFA via autophagy to suppress SAFA-dependent antiviral response. PloS Pathog. 21, e1013201. doi: 10.1371/journal.ppat.1013201 40460159 PMC12132933

[B135] Yu Z. Song H. Jia M. Zhang J. Wang W. Li Q. . (2017). USP1-UAF1 deubiquitinase complex stabilizes TBK1 and enhances antiviral responses. J. Exp. Med. 214, 3553–3563. doi: 10.1084/jem.20170180 29138248 PMC5716033

[B136] Zhang Y. Geng X. Li Q. Xu J. Tan Y. Xiao M. . (2020). m^6^A modification in RNA: biogenesis, functions and roles in gliomas. J. Exp. Clin. Cancer Res. 39, 192. doi: 10.1186/s13046-020-01706-8 32943100 PMC7500025

[B137] Zhang X. Hao H. Ma L. Zhang Y. Hu X. Chen Z. . (2021). Methyltransferase-like 3 modulates severe acute respiratory syndrome coronavirus-2 RNA N6-methyladenosine modification and replication. mBio 12, e0106721. doi: 10.1128/mBio.01067-21 34225491 PMC8437041

[B138] Zhang L. Peng X. Wang Q. Li J. Lv S. Han S. . (2023). CCR2 is a host entry receptor for severe fever with thrombocytopenia syndrome virus. Sci. Adv. 9, eadg6856. doi: 10.1126/sciadv.adg6856 37531422 PMC10396298

[B139] Zhang W. K. Yan J. M. Chu M. Li B. Gu X. L. Jiang Z. Z. . (2025). Bunyavirus SFTSV nucleoprotein exploits TUFM-mediated mitophagy to impair antiviral innate immunity. Autophagy 21, 102–119. doi: 10.1080/15548627.2024.2393067 39189526 PMC11702967

[B140] Zhang X. Yan L. N. Liu B. Y. Zhou C. M. Yu X. J. (2024). The hnRNP A2B1 is important for the replication of SFTSV and other RNA viruses. Microbiol. Spectr 12, e0082924. doi: 10.1128/spectrum.00829-24 39166862 PMC11448443

[B141] Zhang Y. Zhang Q. Li S. Zhao Y. Lv X. Xu X. (2025). Lysozyme (LYZ) promotes PEDV replication via degrading m(6)A methylation of RIG-I transcripts through YTHDF2. Int. J. Biol. Macromol 310, 143297. doi: 10.1016/j.ijbiomac.2025.143297 40253018

[B142] Zhang P. Zhao W. L. Li J. K. Tong J. Y. (2022). RNA m^6^A modification and its roles in immune function regulation. Sichuan Da Xue Xue Bao Yi Xue Ban 53, 1118–1126. doi: 10.12182/20221160511 36443062 PMC10408978

[B143] Zhang J. Zhu Y. Wang X. Wang J. (2023). 25-hydroxycholesterol: an integrator of antiviral ability and signaling. Front. Immunol. 14, 1268104. doi: 10.3389/fimmu.2023.1268104 37781400 PMC10533924

[B144] Zheng Y. Gao C. (2023). Phase separation: The robust modulator of innate antiviral signaling and SARS-CoV-2 infection. Pathogens 12 (2), 243. doi: 10.3390/pathogens12020243 36839515 PMC9962166

[B145] Zhou C. M. Jiang Z. Z. Liu N. Yu X. J. (2024). Current insights into human pathogenic phenuiviruses and the host immune system. Virulence 15, 2384563. doi: 10.1080/21505594.2024.2384563 39072499 PMC11290763

[B146] Zhou P. Wu W. Wei J. Yang Y. Jongkaewwattana A. Xiao Y. . (2025). MARCH6 suppresses Tembusu virus replication by targeting viral NS5 protein for TOLLIP-mediated selective autophagic degradation. J. Virol. 99, e0073525. doi: 10.1128/jvi.00735-25 40511919 PMC12282148

[B147] Zhou C. M. Yu X. J. (2021). Unraveling the underlying interaction mechanism between Dabie bandavirus and innate immune response. Front. Immunol. 12, 676861. doi: 10.3389/fimmu.2021.676861 34122440 PMC8190332

